# Supplementary material for: Induced endometrial trauma (endometrial scratch) in the mid-luteal menstrual cycle phase preceding first cycle IVF/ICSI versus usual IVF/ICSI therapy: study protocol for a randomised controlled trial
Source: BMJ Open. 2018 May 20;8(5):e020755. doi: 10.1136/bmjopen-2017-020755 (PMC5961587; doi:10.1136/bmjopen-2017-020755)
Supplement: Author's manuscript [file bmjopen-2017-020755.draft_revisions.pdf]

BMJ Open

BMJ Open is committed to open peer review. As part of this commitment we make the peer review history of every article we publish publicly available.

When an article is published we post the peer reviewers' comments and the authors' responses online. We also post the versions of the paper that were used during peer review. These are the versions that the peer review comments apply to.

The versions of the paper that follow are the versions that were submitted during the peer review process. They are not the versions of record or the final published versions. They should not be cited or distributed as the published version of this manuscript.

BMJ Open is an open access journal and the full, final, typeset and author-corrected version of record of the manuscript is available on our site with no access controls, subscription charges or pay-per-view fees (http://bmjopen.bmj.com).

If you have any questions on BMJ Open's open peer review process please email info.bmjopen@bmj.com

BMJ Open

Induced Endometrial Trauma (endometrial scratch) in the mid-luteal menstrual cycle phase preceding first cycle IVF/ICSI versus usual IVF/ICSI therapy: Study protocol for a randomised controlled trial

Journal:	*BMJ Open*
Manuscript ID	bmjopen-2017-020755
Article Type:	Protocol
Date Submitted by the Author:	28-Nov-2017

Complete List of Authors:	Pye, Clare; Sheffield Teaching Hospitals NHS Foundation Trust, Obstetrics, Gynaecology & Neonatology<br>Chatters, Robin; University of Sheffield, Health and Related Research<br>Cohen, Judith; The University of Sheffield, School of Health and Related Research (ScHARR)<br>Brian, Kate; Fertility UK<br>Cheong, Ying; University of Southampton, Obstetrics and Gynaecology<br>Laird, Susan; Sheffield Hallam University<br>Mohiyiddeen, Lamiya; Central Manchester University Hospitals NHS Foundation Trust, Department Of Reproductive Medicine<br>Skull, Jonathan; Jessop Wing Hospital<br>Walters, Stephen; University of Sheffield, ScHARR<br>Young, T; The University of Sheffield, ScHARR<br>Metwally, Mostafa; Sheffield Teaching Hospitals NHS Foundation Trust, Obstetrics, Gynaecology & Neonatology

Keywords:	Endometrial Trauma, Endometrial Scratch, IVF, Assisted Conception

SCHOLARONE™
Manuscripts

Title

Induced Endometrial Trauma (endometrial scratch) in the mid-luteal menstrual cycle phase preceding first cycle IVF/ICSI versus usual IVF/ICSI therapy: study protocol for a randomised controlled trial.

Author (*and corresponding author)*:

Clare Pye- RN

Lead Research Nurse/Clinical Trial Co-ordinator – Endometrial Scratch Trial

Rm 32, level 4. Jessop Wing

Sheffield Teaching hospitals NHS Foundation Trust

Tree Root Walk,

Sheffield. S10 2SF

Tel: 0114 2268194

E-mail: clare.pye@sth.nhs.uk

Co-Authors

Robin Chatters- Project Manager

University of Sheffield- Clinical Trials Research Unit

Sheffield-UK

Dr Judith Cohen - Research Fellow/ Assistant Director CTRU - Design, Trials and Statistics (DTS). Research Fellow - The NIHR Research Design Service for Yorkshire & the Humber.

School of Health and Related Research (ScHARR)

University of Sheffield

Sheffield. UK

Ms Kate Brian - Patient & Public Involvement (PPI)

Fertility Network Uk

London. – UK

Professor Ying Cheong - Professor of Reproductive Medicine, Honorary Consultant in Reproductive Medicine and Surgery.

Complete Fertility Centre Southampton

University Hospital Southampton NHS Foundation Trust.

Southampton. UK

Dr Susan Laird – Head of the Department of Biosciences and Chemistry

Biomedical Research Centre

Sheffield Hallam University

Sheffield. UK

For peer review only

For peer review only - http://bmjopen.bmj.com/site/about/guidelines.xhtml

Dr Lamiya Mohiyiddeen - Consultant Gynaecologist
Consultant Gynaecologist
Saint Mary's Hospital - Central Manchester University Hospitals
Manchester. UK

Mr Jonathan Skull – Consultant in Reproductive Medicine
Fertility Unit - Jessop Wing
Sheffield Teaching Hospitals NHS Foundation Trust
Sheffield. UK

Professor Stephen Walters - Trial Statistician
School of Health and Related Research and
NIHR Research Design Service Yorkshire and the Humber
University of Sheffield
Sheffield. UK

Dr Tracey Young - Senior Researcher in Health Economics
Senior Researcher in Health Economics
Health Economics & Design Science
(ScHARR)
University of Sheffield
Sheffield. UK

Mr Mostafa Metwally MD FRCOG - Consultant Gynaecologist and Subspecialist in Reproductive Medicine and Surgery
Fertility Unit - Jessop Wing
Sheffield Teaching Hospitals NHS Foundation Trust
Sheffield. UK

Key words:

Endometrial Scratch

In Vitro Fertilisation

Endometrial Trauma

Assisted Conception

Randomised Controlled Trial

For peer review only - http://bmjopen.bmj.com/site/about/guidelines.xhtml

For peer review only

Abstract

Introduction

Endometrial Trauma commonly known as Endometrial Scratch (ES) has been shown to improve pregnancy rates in women with a history of repeated implantation failure undergoing In Vitro Fertilisation (IVF), with or without Intracytoplasmic Sperm Injection (ICSI). However, the procedure has not yet been fully explored in women having IVF/ICSI for the first time. This study aims to examine the effect of performing an ES in the midluteal phase prior to a first time IVF/ICSI cycle on the chances of achieving a clinical pregnancy and live birth. If ES can influence this success rate there would be a significant cost saving to the NHS through decreasing the number of IVF/ICSI cycles necessary to achieve a pregnancy, increase the practice of single embryo transfer (SET) and consequently have a large impact on risks and costs associated with multiple pregnancies.

Methods & Analysis

This 30 month, UK, multi-centre, parallel group, randomised controlled trial includes a 9 month internal pilot and health economic analysis recruiting 1044 women from 16 UK Fertility Units. It will follow up participants to identify if IVF/ICSI has been successful and live birth has occurred up to 6 weeks post-partum. Primary analysis will be on an intention to treat basis. A sub-study of endometrial samples obtained during the ES will assess the role of immune factors in embryo implantation.

Participants randomised to the intervention group will receive the ES procedure in the mid luteal phase prior to first time IVF/ICSI treatment versus usual IVF/ICSI treatment in the control group, with 1:1 randomisation. The primary outcome is live birth rate (LBR) after completed 24 weeks gestation.

Ethics and dissemination

The South Central – Berkshire NREC has approved the trial protocol. The findings will be submitted to peer- reviewed journals and abstracts will be submitted to relevant national and international conferences.

Trial Registration number: ISRCTN: 23800982

Strengths and limitations of this study

- This will be the largest randomised controlled trial to date assessing the effectiveness and cost effectiveness of performing the ES procedure in women having IVF/ICSI for the first time
- The trial will have the potential to inform the practice of offering this ‘add-on treatment’ as well as the practice of single embryo transfer.
- It will determine whether performing an ES is an acceptable and well tolerated procedure.
- Due to the nature of the intervention it is not possible to blind study participants.

For peer review only - http://bmjopen.bmj.com/site/about/guidelines.xhtml

- Potential difficulty with recruitment if patients are not in equipoise about effectiveness of the ES procedure in first time IVF/ISCI cycles.

Background

The use of local endometrial trauma known as Endometrial Scratch (ES) to improve implantation rates in women undergoing assisted conception was first described in 2003 [1]. The procedure has since been explored in several studies mainly focusing on women with recurrent implantation failure and has been shown to significantly increase pregnancy rates by almost double [2–4]. Three recent systematic reviews have summarised the evidence, however each included different studies. A recent Cochrane review included fourteen randomised studies; seven in women with previous cycle failure, five in an unselected population and one in a first-time cycle [5]. The live birth rate meta-analysis combined trials regardless of the population (i.e. number of previous IVF cycles) and included five studies, reporting a risk ratio (RR) of 1.42 (1.08, 1.85), p=0.02 [6,7]. The odds of achieving a clinical pregnancy were also increased following ES with a RR of 1.34 (1.11, 1.62), p=0.002. The one trial conducted in women undergoing their first IVF cycle indicated the procedure was harmful with an OR of clinical pregnancy rate of 0.30 (0.14, 0.63) p=0.002 [8]. Notably, this trial performed the ES procedure at the time of oocyte retrieval and not in the month prior to the IVF cycle. Despite the concerns around the quality of evidence in using ES and that the trials undertaken so far have been small (most <150 participants), ES has been widely adopted into routine clinical practice in women with recurrent unsuccessful implantation and is currently being provided in some fertility units where women are having IVF/ICSI for the first time [9,10]. Therefore, it is essential that a large well controlled multi-centre trial is conducted to fully investigate the effectiveness and safety of this technique in women undergoing their first cycle of IVF/ICSI.

The Human Fertilisation and Embryology Authority (HFEA) state in their statistical report into multiple births that the risks associated with multiple births is the single biggest health risk associated with fertility treatment [11]. Multiple births carry risks to the health of both the mother and the babies and that birth of a healthy singleton child, born at full term, is therefore the safest outcome of fertility treatment for both mother and child and is best achieved through promoting the practice of single embryo transfer (SET).

If ES can improve the implantation potential of the embryo and therefore improve success rates, ES may encourage an expansion of current SET policies. Inclusion of women with a lower chance of having cryopreserved embryos and a more general increase in the implementation of the practice of SET, could consequently have a large impact on the risks and costs associated with multiple pregnancies as a result of IVF [12].

The exact mechanism by which ES may improve implantation is not yet known, however it is known that implantation is a complex process involving a number of inflammatory mediators including uterine natural killer cells, leukaemia inhibitory factor and interleukin 15 [13]. It is possible that ES may lead to the activation of inflammatory cells such as

For peer review only - http://bmjopen.bmj.com/site/about/guidelines.xhtml

macrophages and dendritic cells, and release of inflammatory mediators such as tumour necrosis factor-α, interleukin-15, growth-regulated oncogene-α and macrophage inflammatory protein 1B [14].

ES has also been shown to cause the modulation of several endometrial genes that may be involved in membrane stability during the process of implantation such as bladder transmembranal protein (UPIb) and adipose differentiation-related protein and mucin 1 [15].

ES is routinely performed as an outpatient procedure. Risks have been identified in a previous study when the procedure was undertaken on the day of oocyte retrieval; however, the procedure is not known to be associated with any particular risks when undertaken in the menstrual cycle preceding that of IVF therapy, apart from period like discomfort whilst performing the procedure [8]. Taking simple analgesics prior to the procedure usually alleviates this. As with any intrauterine procedure there is a potential for intrauterine infection. However women attending for fertility treatment are usually screened for serious vaginal infections such as chlamydia to minimise the risk of any spread of infection when performing the embryo transfer procedure, a similar procedure to an ES as it involves the insertion of a catheter into the uterine cavity.

The main objectives of this trial are to assess the clinical and cost effectiveness of the ES procedure in women aged between 18 and 37 years (inclusive) undergoing their first IVF/ICSI cycle using either antagonist or long protocols to see if it could potentially improve implantation rates and hence encourage the practice of single embryo replacement. A sub-study will be undertaken in two of these centres (Sheffield and Southampton) where endometrial samples obtained from the ES procedure will be stored for later analysis to identify endometrial factors associated with successful pregnancy outcome.

Method and Analysis

The Endometrial Scratch Trial is a multi-centre, parallel group, randomised controlled trial to examine the clinical, cost effectiveness and safety of an ES performed in the mid-luteal phase prior to a first time IVF/ICSI cycle. Eligible participants will be randomised to either the treatment as usual (TAU) arm, consisting of usual IVF treatment, or the intervention arm where ES will be performed followed by usual IVF treatment. The overall study design is illustrated below in the study flow chart (figure 1).

For peer review only - http://bmjopen.bmj.com/site/about/guidelines.xhtml

Figure 1. Study flow chart.

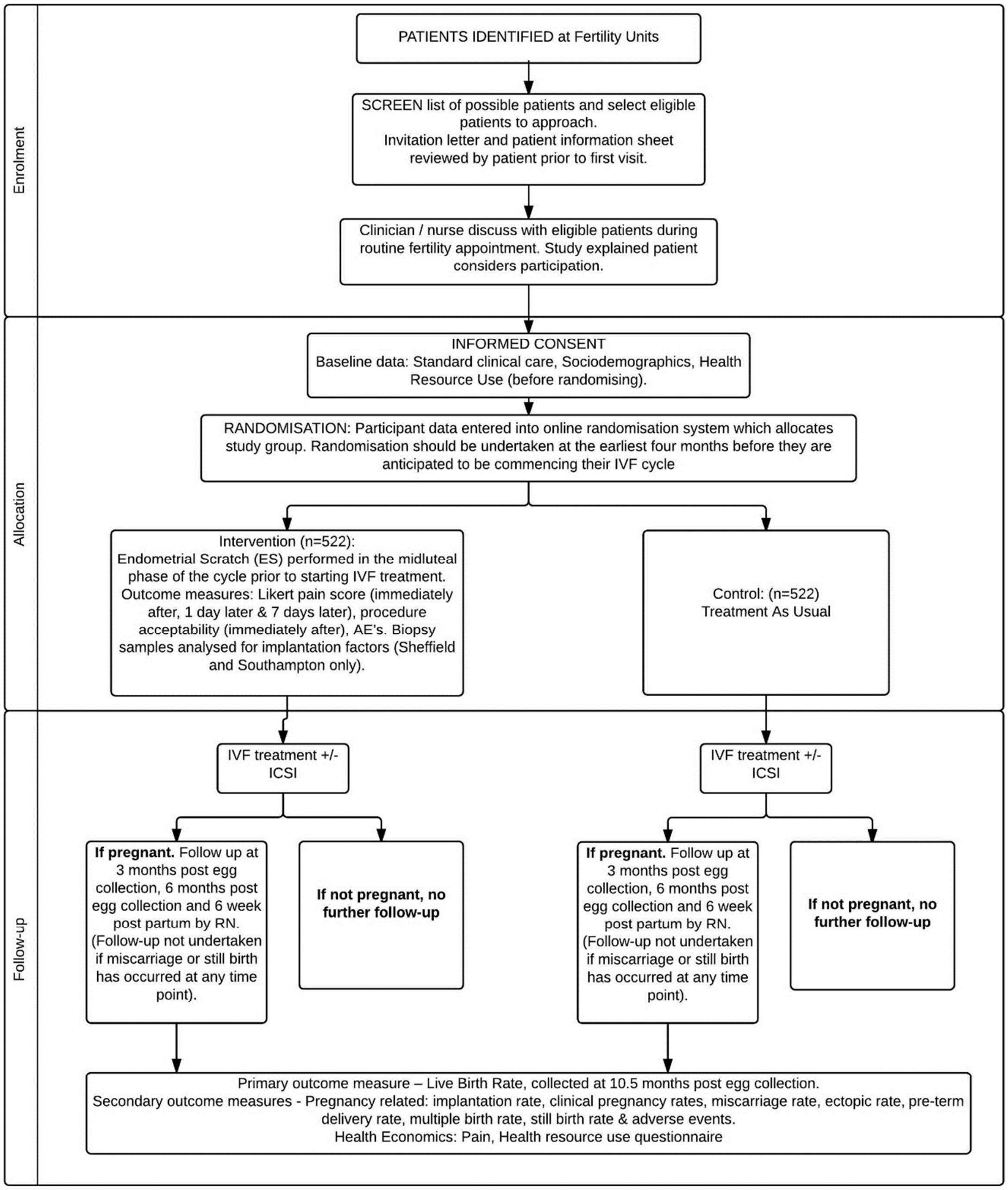

The trial consists of two phases - an internal pilot to assess feasibility of recruitment and delivery of the intervention, and a two year main recruitment phase.

The trial will commence with a 9 month internal pilot recruitment phase across approximately 6 sites to justify whether or not the recruitment strategy and the scheduling of the endometrial scratch procedure are feasible and will use the same trial procedures as described for the main trial.

For peer review only - http://bmjopen.bmj.com/site/about/guidelines.xhtml

The trial is collaboration between research staff at The Jessop Wing, Sheffield Teaching Hospital NHS Foundation Trust & the University of Sheffield - Clinical Trials Research Unit (CTRU) who are responsible for the conduct of the trial. Funding to run the trial has been awarded by the National Institute of Healthy Research (NIHR) Health Technology Assessment (HTA). At the end of the pilot phase, the Trial Steering Committee (TSC) will report to the funder on whether the feasibility criteria have been met and whether the trial should continue.

The trial will be conducted in compliance with the protocol, GCP and regulatory requirements.

Sheffield CTRU will aggregate feasibility of the research and intervention protocols based on the following outcomes.

The trial will be considered infeasible and will be stopped if either of the following conditions apply:

1. Feasibility of recruitment to the main trial: defined as recruitment of fewer than 108 participants (75% of the 144 target) during the internal pilot phase.

2. Scheduling of the ES procedure: defined as less than 75% of women scheduled to receive their ES procedure have received the ES at the correct time point.

Recruitment

Upon successful completion of the pilot the main trial will aim to recruit women attending 16 UK Fertility Units for first time IVF treatment. Participation is entirely voluntary and choosing not to participate will not negatively influence the woman's treatment in any way. Furthermore consent can be withdrawn at any stage. Women who are about to undergo their first cycle of IVF/ICSI will be identified by screening patients referred for these treatments. Eligible women will be sent information regarding the trial in the post or via e-mail or may be alerted to the trial via the trial website or posters displayed at the fertility unit. If they are interested in participating they will be invited to discuss the trial with their fertility team at their next routine appointment.

Prior to randomisation full written informed consent will be obtained by a suitably trained Doctor or Research Nurse/Midwife at a clinic visit. The participant will complete a study specific resource use questionnaire prior to randomisation to collect health care usage in the previous 3 months; baseline data will be collected at this visit and participants will be randomly allocated to either the intervention or usual care arm of the trial.

Detailed methods of the Endometrial Scratch trial are described in the Endometrial Scratch protocol available on the website – https://www.sheffield.ac.uk/scratchtrial

For peer review only - http://bmjopen.bmj.com/site/about/guidelines.xhtml

Women will be included and considered suitable if they meet the following eligibility criteria:

Inclusion criteria

- aged between 18 and 37 years (inclusive) at the time of egg collection and having IVF/ICSI for the 1st time using the antagonist or long protocol only
- expected to receive treatment using fresh embryos and considered to be good responders to treatment (Regular ovulatory menstrual cycle, Normal uterine cavity, expected good ovarian reserve) and where single embryo transfer is expected at the point of entry into the trial.
- no relevant vaginal/uterine infections and are willing to use an appropriate method of barrier contraception (if randomised to Endometrial Scratch in the cycle where the ES procedure is performed), understands and are willing to comply with the trial protocol.

Exlcusion criteria

- previous trauma/surgery to the endometrium and have a BMI of 35 kg/m2 or greater with known grade 4 (severe) endometriosis, are currently participating in any other fertility study involving medical/surgical intervention.
- expected to receive ultra-long protocol, have previously received or have planned an endometrial scratch (or similar procedure, e.g. endometrial biopsy for the collection of Natural Killer Cells) or previously randomised into this trial.

Sampling

The primary outcome is the live birth rate (LBR). This is defined as a live birth after completed 24 weeks gestation, within the 10.5 month post egg collection follow-up period.This time-period will enable the collection of any neonatal deaths (up to 6 weeks post-partum). The denominator for calculating the LBR will be the number of women randomised to each group. Data from the HFEA suggests a live birth rate of 32.8% in women under 35 and 27.3 % in women aged 35-37. The sample size calculation assumes a 30% LBR in the control group and that an absolute increase of 10%, to a 40% LBR (a relative risk of 1.33) in the intervention groups is of clinical and practical importance. The effect size, a 10% absolute difference in LBR, we are proposing is large but we believe an effect of such magnitude is needed to change clinical practice (there is a 5% absolute difference in LBR between women aged under 35 and 35-37) and is less than that observed in the systematic reviews described above (where, at the time of sample size calculation, the relative risk estimates for live birth ranged from 1.83 to 2.46) [2,16]. To have a 90% power of detecting this difference or more, in LBR rates between the groups, as statistically significant at the 5% two-sided level, will require 496 women per group (992 in total). Adjusting for a predicted drop-out rate of 5% (due to anticipated difficulties of follow-up for patients who have been

For peer review only - http://bmjopen.bmj.com/site/about/guidelines.xhtml

referred from NHS Trusts other than the participating Fertility Unit) we will require 1044 participants.

Study procedures

Following randomisation women in the intervention arm will have the ES procedure performed in the midluteal phase of their cycle prior to their planned IVF/ICSI cycle in the outpatient setting of the fertility unit. The choice of screening for infection prior to the procedure or the administration of antibiotics will be left to individual units according to their local established protocols and procedures. Women can be randomised any time up until they start their IVF cycle, although it may be necessary for the participant to delay her IVF if randomised to the intervention arm. This decision to delay should be made and agreed by both the patient and her fertility team before randomisation is undertaken. Women randomised to TAU will continue with their IVF/ICSI as planned and will not receive the ES procedure.

Following delivery of the ES, participants will undergo IVF/ICSI in line with local procedures. Following successful embryo transfer (in both groups) a pregnancy test will be performed and adverse events will be collected. In cases where women do not undergo embryo transfer, every effort will be made by the research team to collect any adverse event information from either the patient or the medical notes. If a pregnancy is confirmed the woman is discharged to normal antenatal care as per standard practice.

Randomisation

The randomisation schedule will be generated by Sheffield CTRU prior to the start of the trial and the randomisation sequence computer generated and stratified by site and protocol (antagonist or long protocol). Random permuted blocks of variable size will be used to ensure enough participants are allocated evenly to each arm of the trial at each site.

Trial Intervention

ES is a minor procedure of 10 to 20 minute duration that will be performed in an outpatient setting at local IVF centres in line with local procedures and the trial standard operation procedure (SOP). The participant will be required to use a barrier method of contraception (if necessary) during the menstrual cycle in which the ES will be performed. During ES, a speculum is inserted into the vagina and the cervix exposed and cleaned. A pipelle or similar endometrial sampler is then inserted into the cavity of the uterus; negative pressure is applied by withdrawal of the plunger. The sampler is rotated and withdrawn several times so that tissue appears in the transparent tube. The sampler and speculum are then removed. If no tissue is seen in the transparent sampler, this is an indication that the sampler was not fully inside the uterine cavity and therefore the procedure is repeated. Following the procedure women will complete a visual pain scale (likert) to assess their pain and tolerability assessment of the procedure within 30 minutes of the initial ES and then again at 24 hours and 7 days post procedure via an automated text message.

For peer review only - http://bmjopen.bmj.com/site/about/guidelines.xhtml

Compliance to the intervention will be ascertained through the clinician or Research Nurse/Midwife recording whether or not the patients has a) attended the clinic for the ES procedure and b) received the ES procedure as per protocol. Any deviation from the protocol will be noted and reported as per the Sheffield CTRU SOP.

Follow-up

Patient follow up will continue until the 1st cycle of IVF or the resulting pregnancy has concluded. If no pregnancy is confirmed the study is complete (regardless of which group the woman is randomised to). Pregnant women will be followed-up at 3 and 6 months post egg collection and then 6 weeks post-partum to collect pregnancy outcome data. If the pregnancy is ongoing at 3 months and 6 week post-partum, a health resource use questionnaire will be sent to the patient for completion. If a spontaneous pregnancy is achieved between randomisation and IVF treatment, the pregnancy will be followed up as described above, instead of egg collection, the date of the last menstrual period (LMP) will be used to schedule the 3 and 6- month follow ups.

Safety considerations, safety monitoring and AE reporting

All Adverse Events (AEs) and Serious Adverse Events (SAE) will be recorded by the local research team at each Fertility Unit. All AEs/SAEs will be followed up until satisfactory resolution or until the treating clinician and the principal investigator deems the event to be chronic or the participant to be stable. Research Nurses/Midwives will ask patients for any details of adverse events at five time-points: post procedure (if randomised to receive ES), at the participants' pregnancy test, and then, if pregnancy has been achieved, at 3 and 6 months post egg collection and finally 6 weeks post-partum.

AEs/SAEs will be collected up to the participants' final study related follow-up event. If embryo transfer does not occur, the Research Nurse/Midwife will contact the participant approximately 2 weeks after egg collection to identify if any adverse events have occurred. In the case of a negative pregnancy test, the site research team should make every effort to obtain AE data from the patient or the medical notes at routine clinical care contacts; no further contact will be made outside of routine clinical care.

Expected AEs will be those which occur regularly due to pregnancy, and expected SAEs are those events which are expected in the patient population as a result of the routine care/treatment of a patient. Expected SAEs and all AEs will be collected as part of the trial and entered into the eCRF, but will not be reported to regulatory bodies (NHS REC/sponsor). Unexpected SAEs will be reported to the Sheffield CTRU as soon as staff at the fertility unit becomes aware of the event.

All SAEs will be reviewed by the Data Monitoring & Ethics Committee (DMEC) and Trial Management Group (TMG) at regular intervals. The Chief Investigator (CI) will inform all Principal Investigators (PIs) concerned of relevant information that would adversely affect the safety of the participants.

For peer review only - http://bmjopen.bmj.com/site/about/guidelines.xhtml

Outcomes

Primary clinical outcome

- Live birth rate; based on the number of live births after 24 weeks gestation within the 10.5 month post egg collection follow-up period.

Secondary outcomes

- Acceptability and pain rating of the Endometrial Scratch procedure, a visual pain scale (likert) to assess their pain and tolerability assessment of the procedure within 30minutes of the initial ES procedure, 1 day later and then again 7 days after the ES.
- Implantation rate
- Clinical pregnancy rate
- Miscarriage rate
- Ectopic pregnancy rate
- Multiple birth rate
- Preterm delivery rate
- Still birth rate
- Details of participant's IVF cycle (fertilisation, egg collection and embryo quality & transfer)
- Adverse events
- Health resource use of the participant & patient costs

The trial includes a health economic component to assess the cost of the intervention per extra live birth from an NHS and social care perspective. Resource use will include the intervention costs for ES, the cost of IVF treatment, visits to the assisted conception unit and for those who conceive antenatal and post-natal visits, delivery costs and any hospital stays not related to birth for both mother and baby. The resource use questionnaire will collect information on contacts with midwife and GP visits. A Patient Cost questionnaire will collect time taken to travel to appointments and loss of productivity. Unit costs will be derived from appropriate national sources and will include; NHS reference costs, Personal Social Service Research Unit costs and the **Office of National Statistics** [17–19]. The resource use questionnaire will be designed for this study and will draw on data collection tools developed in The School of Health and Related Research (ScHARR) and those collated by the Database for Instruments for Resource Use Measurement (DIRUM).

Blinding

Due to the nature of the intervention, it will not be possible to blind patients or clinicians to treatment allocation and since this trial evaluates objectively measured outcomes (pregnancy rates) that are unlikely to be affected by a placebo effect, it is not necessary to perform a sham procedure for the control group. The study statistician, TSC and health economist will be blinded to the allocation.

For peer review only - http://bmjopen.bmj.com/site/about/guidelines.xhtml

Trial monitoring and oversight committees

The trial will be overseen by the TSC and the DMEC, membership of both will consist of independent experts in the field. The TSC will include a patient representative. Both committees will review recruitment, study progress and adverse events. The DMEC will receive monthly reports of recruitment and adverse events and, at their meetings, will also consider emerging evidence from other trials or research on ES. They may advise the chair of the TSC at any time if, in their view, the trial should be stopped for ethical reasons, including concerns about patient safety.

Day-to-day running of the trial will be coordinated by the Trial Management Group (TMG), consisting of the grant co-applicants, plus members of the Jessop Wing Fertility Unit, Sheffield CTRU and patient representatives.

Statistical analysis

Primary analysis will be performed on the intention to treat population (all participants randomised into the trial). All statistical exploratory tests will be two-tailed at 5% nominal level. Baseline demographic (e.g. age), physical measurements (e.g. BMI), and health-related data will be described and summarised overall and for both treatment groups. The women, not the IVF cycle will be the unit of analysis. If the woman fails to get pregnant or does not have IVF treatment, they will be included in the analysis of the primary outcome as a negative outcome (i.e. non-live birth). For sensitivity analyses, per protocol (PP) analyses will also be undertaken which will be defined as for Endometrial Scratch participants in the intervention group, receiving the ES procedure as documented in the study protocol and undergoing IVF/ICSI in the subsequent menstrual cycle, including embryo transfer. For the control group, the PP population will receive IVF/ICSI including embryo transfer. Sub-group analyses will be undertaken to explore the effect of important variables related to the participant and their treatment on the primary and secondary outcomes. These subgroups are:

- Day of embryo transfer (day 2, 3, 4, 5 or 6),
- Fertilisation method (IVF, IVF or ICSI, ICSI [spilt]),
- Type of protocol (long or antagonistic),
- Embryo transfer (single or double) and whether the embryo was fresh or frozen
- Previous history of consecutive miscarriages (0-2 vs >=3)

AEs will be reported as a proportion of all women randomised. Adverse events including serious adverse events will be compared between the two groups using a Fisher's Exact test, Chi-squared test or negative binomial regression model in case of repeated events per woman (as appropriate). A 95% CI for the difference in adverse event rate between the

For peer review only - http://bmjopen.bmj.com/site/about/guidelines.xhtml

groups will also be calculated with associated point estimate depending on the method used.

Health economic results will be presented in the net-benefit framework and will allow for uncertainty using bootstrapping and probabilistic sensitivity analysis.

Ethics and dissemination

The study is registered on the ISRCTN database (reference 23800982) and has been approved by the South Berkshire Research Ethics Committee (reference 16/SC/0151). The findings of this trial will be submitted to peer- reviewed journals and abstracts to national and international conferences. Other stakeholder specific outputs in relevant formats will also be produced for commissioners, IVF practitioners, third sector and user advocacy organisations. A website will be established to promote the work of the trial. All knowledge transfer activity including translation will be informed by input from trial collaborators, the TSC and TMG to ensure the study is meeting the needs of the commissioners and audience.

Discussion

This trial will determine whether performing an ES procedure prior to 1st time IVF/ICSI treatment is an inexpensive, safe and well tolerated procedure that increases the live birth rate in women having SET. If shown to be the case, this will have a significant improvement in first cycle IVF success rates and potentially lead to significant cost savings to the NHS as fewer women would need to have repeat treatment cycles. This is particularly important in the current economic climate and with restrictions on funding and service provision. This will also have a significant impact for women, for whom the burden of repeated cycles is large.

Acknowledgements

We thank Dr Munya Dimairo - Research Fellow in Medical Statistics at the University of Sheffield for his contribution to the design/content of the protocol, The Jessop Wing Reproductive Health Public Advisory Panel as well as each local PI, Research Nurse/Midwife and research delivery team in the recruitment and management of patients.

Authors Contributions

Contributors: MM, CP, JC, KB, YC, SL, LM, JS, SW &, TY conceived the study, and contributed to study design, sample size calculations and analytical plans.

MM, CP, RC, JC, KB, YC, SL, LM, JS, SW & TY initiated the project, have assisted in developing the protocol and helped with implementation.

CP, RC, JC, & MM drafted the manuscript. All authors read and approved the final manuscript.

Competing interests

None declared.

For peer review only - http://bmjopen.bmj.com/site/about/guidelines.xhtml

Funding statement

This trial was funded by the NIHR Health Technology Assessment program grant number [14/08/45]

This article presents independent research supported by the National Institute for Health Research (NIHR). The views expressed are those of the author(s) and not necessarily those of the NHS, the NIHR or the Department of Health

References

1 Barash A, Dekel N, Fieldust S, *et al.* Local injury to the endometrium doubles the incidence of successful pregnancies in patients undergoing in vitro fertilization. *Fertil Steril* 2003;**79**:1317–22. doi:10.1016/S0015-0282(03)00345-5

2 El-Toukhy T, Sunkara S, Khalaf Y. Local endometrial injury and IVF outcome: A systematic review and meta-analysis. *Reprod Biomed Online* 2012;**25**:345–54. doi:10.1016/j.rbmo.2012.06.012

3 Raziel A, Schachter M, Strassburger D, *et al.* Favorable influence of local injury to the endometrium in intracytoplasmic sperm injection patients with high-order implantation failure. *Fertil Steril* 2007;**87**:198–201. doi:10.1016/j.fertnstert.2006.05.062

4 Huang SY, Wang C-J, Soong Y-K, *et al.* Site-specific endometrial injury improves implantation and pregnancy in patients with repeated implantation failures. *Reprod Biol Endocrinol* 2011;**9**:140. doi:10.1186/1477-7827-9-140

5 Nastri CO, Lensen SF, Gibreel A, *et al.* Endometrial injury in women undergoing assisted reproductive techniques. In: Martins WP, ed. *Cochrane Database of Systematic Reviews*. Chichester, UK: : John Wiley & Sons, Ltd 2015. doi:10.1002/14651858.CD009517.pub3

6 Narvekar S a, Gupta N, Shetty N, *et al.* Does local endometrial injury in the nontransfer cycle improve the IVF-ET outcome in the subsequent cycle in patients with previous unsuccessful IVF? A randomized controlled pilot study. *J Hum Reprod Sci* 2010;**3**:15–9. doi:10.4103/0974-1208.63116

7 The effect of local injury to the endometrium for implantation and pregnancy rates in ICSI -ET cycles with recurrent implantation failure: a randomised controlled study. 2011.clinicaltrials.gov/NCT01340560

8 Karimzade MA, Oskouian H, Ahmadi S, *et al.* Local injury to the endometrium on the day of oocyte retrieval has a negative impact on implantation in assisted reproductive cycles: A randomized controlled trial. *Arch Gynecol Obstet* 2010;**281**:499–503. doi:10.1007/s00404-009-1166-1

9 Spencer EA, Mahtani KR, Goldacre B, *et al.* Claims for fertility interventions: a systematic assessment of statements on UK fertility centre websites. *BMJ Open* 2016;**6**:e013940. doi:10.1136/bmjopen-2016-013940

For peer review only - http://bmjopen.bmj.com/site/about/guidelines.xhtml

10 Lensen S, Sadler L, Farquhar C. Endometrial scratching for subfertility: Everyone's doing it. *Hum Reprod* 2016;**31**:1241–4. doi:10.1093/humrep/dew053

11 Human Fertilisation & Embryology Authority. Improving outcomes for fertility patients: Multiple births. A statistical report. 2011.

12 Ledger WL, Anumba D, Marlow N, *et al.* The costs to the NHS of multiple births after IVF treatment in the UK. *BJOG* 2006;**113**:21–5. doi:10.1111/j.1471-0528.2005.00790.x

13 Tuckerman E, Mariee N, Prakash A, *et al.* Uterine natural killer cells in peri-implantation endometrium from women with repeated implantation failure after IVF. *J Reprod Immunol* 2010;**87**:60–6. doi:10.1016/j.jri.2010.07.001

14 Gnainsky Y, Granot I, Aldo PB, *et al.* Local injury of the endometrium induces an inflammatory response that promotes successful implantation. *Fertil Steril* 2010;**94**:2030–6. doi:10.1016/j.fertnstert.2010.02.022

15 Kalma Y, Granot I, Gnainsky Y, *et al.* Endometrial biopsy-induced gene modulation: first evidence for the expression of bladder-transmembranal uroplakin Ib in human endometrium. *Fertil Steril* 2009;**91**:1042–9, 1049-9. doi:10.1016/j.fertnstert.2008.01.043

16 Nastri CO, Gibreel A, Raine-Fenning N, *et al.* Endometrial injury in women undergoing assisted reproductive techniques. *Cochrane database Syst Rev* 2012;**7**:CD009517. doi:10.1002/14651858.CD009517.pub2

17 NHS reference costs 2012/13. 2013.

18 Curtis L. Unit costs of health and social care 2013. 2013.

19 Annual survey for hours and earnings (ASHE). 2012. http://www.ons.gov.uk/ons/rel/ashe/annual-survey-of-hours-and-earnings/2012-revised-results/index.html (accessed 12 Sep 2014).

For peer review only

For peer review only - http://bmjopen.bmj.com/site/about/guidelines.xhtml

BMJ Open

Induced Endometrial Trauma (endometrial scratch) in the mid-luteal menstrual cycle phase preceding first cycle IVF/ICSI versus usual IVF/ICSI therapy: Study protocol for a randomised controlled trial.

Journal:	*BMJ Open*
Manuscript ID	bmjopen-2017-020755.R1
Article Type:	Protocol
Date Submitted by the Author:	18-Jan-2018

Complete List of Authors:	Pye, Clare; Sheffield Teaching Hospitals NHS Foundation Trust, Obstetrics, Gynaecology & Neonatology<br>Chatters, Robin; University of Sheffield, Health and Related Research<br>Cohen, Judith; The University of Sheffield, School of Health and Related Research (ScHARR)<br>Brian, Kate; Fertility UK<br>Cheong, Ying; University of Southampton, Obstetrics and Gynaecology<br>Laird, Susan; Sheffield Hallam University<br>Mohiyiddeen, Lamiya; Central Manchester University Hospitals NHS Foundation Trust, Department Of Reproductive Medicine<br>Skull, Jonathan; Jessop Wing Hospital<br>Walters, Stephen; University of Sheffield, ScHARR<br>Young, T; The University of Sheffield, ScHARR<br>Metwally, Mostafa; Sheffield Teaching Hospitals NHS Foundation Trust, Obstetrics, Gynaecology & Neonatology

**Primary Subject Heading**:	Reproductive medicine
Secondary Subject Heading:	Obstetrics and gynaecology
Keywords:	Endometrial Trauma, Endometrial Scratch, IVF, Assisted Conception

SCHOLARONE™
Manuscripts

Title

Induced Endometrial Trauma (endometrial scratch) in the mid-luteal menstrual cycle phase preceding first cycle IVF/ICSI versus usual IVF/ICSI therapy:
Study protocol for a randomised controlled trial.

Author (*and corresponding author)*:
Clare Pye- RN
Lead Research Nurse/Clinical Trial Co-ordinator – Endometrial Scratch Trial
Rm 32, level 4. Jessop Wing
Sheffield Teaching hospitals NHS Foundation Trust
Tree Root Walk,
Sheffield. S10 2SF
Tel: 0114 2268194
E-mail: clare.pye@sth.nhs.uk

Co-Authors

Robin Chatters- Trial Manager
University of Sheffield- Clinical Trials Research Unit
Sheffield-UK

Dr Judith Cohen - Research Fellow/ Assistant Director CTRU - Design, Trials and Statistics (DTS). Research Fellow - The NIHR Research Design Service for Yorkshire & the Humber.
School of Health and Related Research (ScHARR)
University of Sheffield
Sheffield.
UK

Ms Kate Brian - Patient & Public Involvement (PPI)
Fertility Network Uk
London.
UK

Professor Ying Cheong - Professor of Reproductive Medicine, Honorary Consultant in Reproductive Medicine and Surgery.
Complete Fertility Centre Southampton
University Hospital Southampton NHS Foundation Trust.
Southampton.
UK

For peer review only
For peer review only - http://bmjopen.bmj.com/site/about/guidelines.xhtml

Dr Susan Laird – Head of the Department of Biosciences and Chemistry
Biomedical Research Centre
Sheffield Hallam University
Sheffield.
UK

Dr Lamiya Mohiyiddeen - Consultant Gynaecologist and Subspecialist in Reproductive Medicine and Surgery
Saint Mary's Hospital - Central Manchester University Hospitals
Manchester.
UK

Mr Jonathan Skull – Consultant in Reproductive Medicine
Fertility Unit - Jessop Wing
Sheffield Teaching Hospitals NHS Foundation Trust
Sheffield.
UK

Professor Stephen Walters - Trial Statistician
School of Health and Related Research and
NIHR Research Design Service Yorkshire and the Humber
University of Sheffield
Sheffield.
UK

Dr Tracey Young - Senior Researcher in Health Economics
Senior Researcher in Health Economics
Health Economics & Design Science
(ScHARR)
University of Sheffield
Sheffield.
UK

Mr Mostafa Metwally MD FRCOG - Consultant Gynaecologist and Subspecialist in Reproductive Medicine and Surgery
Fertility Unit - Jessop Wing
Sheffield Teaching Hospitals NHS Foundation Trust
Sheffield.
UK

For peer review only

For peer review only - http://bmjopen.bmj.com/site/about/guidelines.xhtml

Key words:

Endometrial Scratch

In Vitro Fertilisation

Endometrial Trauma

Assisted Conception

Randomised Controlled Trial

Abstract

Introduction
Endometrial Trauma commonly known as Endometrial Scratch (ES) has been shown to improve pregnancy rates in women with a history of repeated implantation failure undergoing In Vitro Fertilisation (IVF), with or without Intracytoplasmic Sperm Injection (ICSI). However, the procedure has not yet been fully explored in women having IVF/ICSI for the first time. This study aims to examine the effect of performing an ES in the midluteal phase prior to first time IVF/ICSI cycle on the chances of achieving a clinical pregnancy and live birth. If ES can influence this success rate there would be a significant cost saving to the NHS through decreasing the number of IVF/ICSI cycles necessary to achieve a pregnancy, increase the practice of single embryo transfer (SET) and consequently have a large impact on risks and costs associated with multiple pregnancies.

Methods & Analysis
This 30 month, UK, multi-centre, parallel group, RCT includes a 9month internal pilot and health economic analysis recruiting 1044 women from 16 Fertility Units. It will follow up participants to identify if IVF/ICSI has been successful and live birth has occurred up to 6 weeks post-partum. Primary analysis will be on an intention to treat basis. A sub-study of endometrial samples obtained during the ES will assess the role of immune factors in embryo implantation. Main trial recruitment commenced January 2017 and is ongoing.

Participants randomised to the intervention group will receive the ES procedure in the mid luteal phase prior to first time IVF/ICSI treatment versus usual IVF/ICSI treatment in the control group, with 1:1 randomisation. The primary outcome is live birth rate (LBR) after completed 24 weeks gestation.

Ethics and dissemination
South Central – Berkshire NREC approved the protocol. Findings will be submitted to peer-reviewed journals and abstracts will be submitted to relevant national and international conferences.

For peer review only - http://bmjopen.bmj.com/site/about/guidelines.xhtml

Trial Registration number: ISRCTN: 23800982. Protocol Date: Version 5 Dated 20/07/2017

Strengths and limitations of this study

- This is the largest multicentre, pragmatic randomised controlled trial to date which aims to assess the effectiveness and cost effectiveness of performing the ES procedure in women having IVF/ICSI for the first time
- It aims to determine whether performing an ES is an acceptable and well tolerated procedure.
- Due to the nature of the intervention it is not possible to blind study participants or clinicians.
- Potential difficulty with recruitment if patients are not in equipoise about effectiveness of the ES procedure in first time IVF/ISCI cycles.

Background

The use of local endometrial trauma known as Endometrial Scratch (ES) to improve implantation rates in women undergoing assisted conception was first described in 2003 [1]. The procedure has since been explored in several studies mainly focusing on women with recurrent implantation failure and has been shown to significantly increase pregnancy rates by almost double [2–4]. However, uncertainty remains as to the therapeutic effect of ES, due to heterogeneity of the populations included - and the timing and exact protocol of ES used - in previous evaluations [5,6]. Three systematic reviews have summarised the evidence, however each included different studies [2,7,8]. A recent Cochrane review included fourteen randomised studies; seven in women with previous cycle failure, five in an unselected population and one in a first-time cycle [8]. The live birth rate meta-analysis combined trials regardless of the population (i.e. number of previous IVF cycles) and included five studies, reporting a risk ratio (RR) of 1.42 (1.08, 1.85), p=0.02. The odds of achieving a clinical pregnancy were also increased following ES with a RR of 1.34 (1.11, 1.62), p=0.002. The one trial conducted in women undergoing their first IVF cycle indicated the procedure was harmful with an OR of clinical pregnancy rate of 0.30 (0.14, 0.63) p=0.002 [9]. Notably, this trial performed the ES procedure at the time of oocyte retrieval and not in the month prior to the IVF cycle. Despite the concerns around the quality of evidence in using ES and that many of the trials undertaken so far have been small (most <150 participants), ES has been widely adopted into routine clinical practice in women with recurrent unsuccessful implantation and is currently being provided in some fertility units where women are having IVF/ICSI for the first time [10,11]. Two large trials are currently in progress to determine if ES is beneficial in women undergoing their 2nd IVF cycle [12] and a sample of women undergoing any IVF cycle [13]. Therefore, given the lack of evidence for the effectiveness of ES in women undergoing their 1st cycle of IVF/ICSI, it is essential that a large well controlled multi-centre trial is conducted to fully investigate the effectiveness and safety of this technique.

For peer review only - http://bmjopen.bmj.com/site/about/guidelines.xhtml

The Human Fertilisation and Embryology Authority (HFEA) state in their statistical report into multiple births that the risks associated with multiple births is the single biggest health risk associated with fertility treatment [14]. Multiple births carry risks to the health of both the mother and the babies and that birth of a healthy singleton child, born at full term, is therefore the safest outcome of fertility treatment for both mother and child and is best achieved through promoting the practice SET.

If ES can improve the implantation potential of the embryo and therefore improve success rates, ES may encourage an expansion of current SET policies. Inclusion of women with a lower chance of having cryopreserved embryos and a more general increase in the implementation of the practice of SET, could consequently have a large impact on the risks and costs associated with multiple pregnancies as a result of IVF [15].

The exact mechanism by which ES may improve implantation is not yet known, however it is known that implantation is a complex process involving a number of inflammatory mediators including uterine natural killer cells, leukaemia inhibitory factor and interleukin 15 [13]. It is possible that ES may lead to the activation of inflammatory cells such as macrophages and dendritic cells, and release of inflammatory mediators such as tumour necrosis factor-α, interleukin-15, growth-regulated oncogene-α and macrophage inflammatory protein 1B [14].

ES has also been shown to cause the modulation of several endometrial genes that may be involved in membrane stability during the process of implantation such as bladder transmembranal protein (UPIb) and adipose differentiation-related protein and mucin 1 [16].

ES is routinely performed as an outpatient procedure. Risks have been identified in a previous study when the procedure was undertaken on the day of oocyte retrieval (reduced implantation and pregnancy rates) [9]; however, the procedure is not known to be associated with any particular risks when undertaken in the menstrual cycle preceding that of IVF therapy, apart from period like discomfort whilst performing the procedure. Taking simple analgesics prior to the procedure usually alleviates this. As with any intrauterine procedure there is a potential for intrauterine infection. However women attending for fertility treatment are usually screened for serious vaginal infections such as chlamydia to minimise the risk of any spread of infection when performing the embryo transfer procedure, a similar procedure to an ES as it involves the insertion of a catheter into the uterine cavity.

The main objectives of this trial are to assess the clinical and cost effectiveness of the ES procedure in women aged between 18 and 37 years (inclusive) undergoing their first IVF/ICSI cycle using either antagonist or long protocols to see if it could potentially improve implantation rates and hence encourage the practice of single embryo replacement. A sub-study will be undertaken in two of these fertility units) where endometrial samples obtained

For peer review only - http://bmjopen.bmj.com/site/about/guidelines.xhtml

from the ES procedure will be stored for later analysis to identify endometrial factors associated with successful pregnancy outcome.

Method and Analysis

The Endometrial Scratch Trial is a multi-centre, parallel group, randomised controlled trial to examine the clinical, cost effectiveness and safety of an ES performed in the mid-luteal phase prior to a first time IVF/ICSI cycle. Eligible participants will be randomised to either the treatment as usual (TAU) arm, consisting of usual IVF treatment, or the intervention arm where ES will be performed followed by usual IVF treatment. The overall study design is illustrated below in the study flow chart (figure 1).

Figure 1. Study flow chart.

The trial consists of two phases - an internal pilot to assess feasibility of recruitment and delivery of the intervention, and a two year main recruitment phase.

The trial will commence with a 9 month internal pilot recruitment phase across approximately 6 UK fertility units to justify whether or not the recruitment strategy and the scheduling of the endometrial scratch procedure are feasible and will use the same trial procedures as described for the main trial.

The trial is collaboration between research staff at The Jessop Wing, Sheffield Teaching Hospital NHS Foundation Trust & the University of Sheffield - Clinical Trials Research Unit (CTRU) who are responsible for the conduct of the trial. Funding to run the trial has been awarded by the National Institute of Healthy Research (NIHR) Health Technology Assessment (HTA). At the end of the pilot phase, the Trial Steering Committee (TSC) will report to the funder on whether the feasibility criteria have been met and whether the trial should continue.
The trial will be conducted in compliance with the protocol, GCP and regulatory requirements. Main trial recruitment commenced January 2017 and is ongoing.

Sheffield CTRU will aggregate feasibility of the research and intervention protocols based on the following outcomes.
The trial will be considered infeasible and will be stopped if either of the following conditions apply:

1. Feasibility of recruitment to the main trial: defined as recruitment of fewer than 108 participants (75% of the 144 target) during the internal pilot phase.

2. Scheduling of the ES procedure: defined as less than 75% of women scheduled to receive their ES procedure have received the ES at the correct time point.

For peer review only - http://bmjopen.bmj.com/site/about/guidelines.xhtml

Recruitment

Upon successful completion of the pilot the main trial will aim to recruit women attending 16 UK Fertility Units for first time IVF treatment. Participation is entirely voluntary and choosing not to participate will not negatively influence the woman's treatment in any way. Furthermore consent can be withdrawn at any stage. Women who are about to undergo their first cycle of IVF/ICSI will be identified by screening patients referred for these treatments. Eligible women will be sent information regarding the trial in the post or via e-mail or may be alerted to the trial via the trial website or posters displayed at the fertility unit. If they are interested in participating they will be invited to discuss the trial with their fertility team at their next routine appointment.

Prior to randomisation full written informed consent will be obtained by a suitably trained Doctor or Research Nurse/Midwife at a clinic visit. The participant will complete a study specific resource use questionnaire prior to randomisation to collect health care usage in the previous 3 months; baseline data will be collected at this visit and participants will be randomly allocated to either the intervention or usual care arm of the trial.

Detailed methods of the Endometrial Scratch trial are described in the Endometrial Scratch protocol available on the website – https://www.sheffield.ac.uk/scratchtrial

Women will be included and considered suitable if they meet the following eligibility criteria:

Inclusion criteria

1. Women expected to be aged between 18 and 37 years (inclusive) at time of egg collection.
2. First time IVF with or without ICSI treatment using the antagonist or long protocol only
3. Expected to receive treatment using fresh embryos.
4. Expected good responders to treatment, with:
    a. Ovulatory menstrual cycle (Regular menstrual cycles defined by clinical judgement or with ovulatory levels of midluteal serum progesterone as defined by local laboratory protocols)
    b. Normal uterine cavity (assessed by transvaginal sonography at screening and no endometrial abnormalities such as , suspected intrauterine adhesions, uterine septa, submucosal fibroids or intramural fibroids exceeding 4 cm in diameter as assessed by the investigator that would require treatment to facilitate pregnancy).
    c. Expected good ovarian reserve (assessed clinically, biochemically (FSH< 10 & normal follicular phase oestradiol levels and or normal AMH), and or sonographically (antral follicle counts) and no history of previous radiotherapy or chemotherapy). [All laboratory/ultrasound standards based on local normal reference ranges.]
    d. Single embryo transfer (SET) expected.
5. Local procedures have been / will be followed to exclude relevant vaginal/uterine infections prior to starting treatment.

For peer review only - http://bmjopen.bmj.com/site/about/guidelines.xhtml

6. Willing to use an appropriate method of barrier contraception if randomised to Endometrial Scratch (ES) in the cycle where the ES procedure is performed. .
7. Understands/willing to comply with the protocol.

Exlcusion criteria

1. Previous trauma/surgery to the endometrium (e.g. resection of submucous fibroid, intrauterine adhesions.)
2. BMI of 35 kg/m2 or greater
3. Known grade 4 (severe) endometriosis
4. Currently participating in any other fertility study involving medical/surgical intervention
5. Expected to receive protocols other than antagonist or long (e.g. ultra-long protocol)
6. An endometrial scratch (or similar procedure, e.g. endometrial biopsy for the collection of Natural Killer Cells) is planned
7. Previously randomised into this trial

Sampling

The primary outcome is the LBR. This is defined as a live birth after completed 24 weeks gestation, within the 10.5 month post egg collection follow-up period.This time-period will enable the collection of any neonatal deaths (up to 6 weeks post-partum). The denominator for calculating the LBR will be the number of women randomised to each group. Data from the HFEA suggests a live birth rate of 32.8% in women under 35 and 27.3 % in women aged 35-37. The sample size calculation assumes a 30% LBR in the control group and that an absolute increase of 10%, to a 40% LBR (a relative risk of 1.33) in the intervention groups is of clinical and practical importance. The effect size, a 10% absolute difference in LBR, we are proposing is large but we believe an effect of such magnitude is needed to change clinical practice (there is a 5% absolute difference in LBR between women aged under 35 and 35-37) and is less than that observed in the systematic reviews described above (where, at the time of sample size calculation, the relative risk estimates for live birth ranged from 1.83 to 2.46) [2,17]. To have a 90% power of detecting this difference or more, in LBR rates between the groups, as statistically significant at the 5% two-sided level, will require 496 women per group (992 in total). Adjusting for a predicted drop-out rate of 5% (due to anticipated difficulties of follow-up for patients who have been referred from NHS Trusts other than the participating Fertility Unit) we will require 1044 participants.

Study procedures

Following randomisation women in the intervention arm will have the ES procedure performed in the midluteal phase of their cycle prior to their planned IVF/ICSI cycle in the outpatient setting of the fertility unit. The choice of screening for infection prior to the procedure or the administration of antibiotics will be left to individual units according to their local established protocols and procedures. Women can be randomised any time up until they start their IVF cycle, although it may be necessary for the participant to delay her

For peer review only - http://bmjopen.bmj.com/site/about/guidelines.xhtml

IVF if randomised to the intervention arm. This decision to delay should be made and agreed by both the patient and her fertility team before randomisation is undertaken. The oral contraceptive pill can be used following randomisation for the purposes of cycle programming but women must be having ovulatory periods at the point of entry into the trial. Women randomised to TAU will continue with their IVF/ICSI as planned and will not receive the ES procedure.

Following delivery of the ES, participants will undergo IVF/ICSI in line with local procedures. Following successful embryo transfer (in both groups) a pregnancy test will be performed and adverse events will be collected. In cases where women do not undergo embryo transfer, every effort will be made by the research team to collect any adverse event information from either the patient or the medical notes. If a pregnancy is confirmed the woman is discharged to normal antenatal care as per standard practice. It is the intention to obtain the pregnancy status of all women once randomised including the outcome of all spontaneous pregnancies, the first frozen embryo transfer if no fresh transfer has been undertaken as well as those that delay treatment following the ES. Women will not be followed-up if they withdraw their consent from the trial. Data will also be collected regarding participants who have received an ES outside the trial.

Randomisation

The randomisation schedule will be generated by Sheffield CTRU prior to the start of the trial; access to the schedule will be limited only to the trial statistician. The randomisation sequence will be computer generated and stratified by site and protocol (antagonist or long protocol). Random permuted blocks of variable size will be used to ensure enough participants are allocated evenly to each arm of the trial at each site. Research staff at recruiting centres will be unable to access the randomisation sequence and will use a web based computer system with restricted access rights to enter participant details; randomisation outcome will then be revealed. Re-randomisation will not be permitted.

Trial Intervention

ES is a minor procedure of 10 to 20 minute duration that will be performed in an outpatient setting at local IVF centres in line with local procedures and the trial standard operation procedure (SOP). The participant will be required to use a barrier method of contraception (if necessary) during the menstrual cycle in which the ES will be performed. During ES, a speculum is inserted into the vagina and the cervix exposed and cleaned. A pipelle or similar endometrial sampler is then inserted into the cavity of the uterus; negative pressure is applied by withdrawal of the plunger. The sampler is rotated and withdrawn several times so that tissue appears in the transparent tube. The sampler and speculum are then removed. If no tissue is seen in the transparent sampler, this is an indication that the sampler was not fully inside the uterine cavity and therefore the procedure is repeated. Following the procedure women will complete a visual pain scale (likert) to assess their pain

For peer review only - http://bmjopen.bmj.com/site/about/guidelines.xhtml

and tolerability assessment of the procedure within 30 minutes of the initial ES and then again at 24 hours and 7 days post procedure via an automated text message.

Compliance to the intervention will be ascertained through the clinician or Research Nurse/Midwife recording whether or not the patient has a) attended the clinic for the ES procedure and b) received the ES procedure as per protocol. Any deviation from the protocol will be noted and reported as per the Sheffield CTRU SOP.

Follow-up
Patient follow up will continue until either the 1st cycle of IVF has been completed or the resulting pregnancy has concluded. If no pregnancy is confirmed the study is complete (regardless of which group the woman is randomised to). Pregnant women will be followed-up at 3 and 6 months post egg collection and then 6 weeks post-partum to collect pregnancy outcome and safety data. If the pregnancy is ongoing at 3 months and 6 week post-partum, a health resource use questionnaire will be sent to the patient for completion. If a spontaneous pregnancy is achieved between randomisation and IVF treatment, the pregnancy will be followed up as described above, instead of egg collection, the date of the last menstrual period (LMP) will be used to schedule the 3/6 month & 6 week post-partum follow ups.

Safety considerations, safety monitoring and AE reporting
All Adverse Events (AEs) and Serious Adverse Events (SAE) will be recorded by the local research team at each Fertility Unit. All AEs/SAEs will be followed up until satisfactory resolution or until the treating clinician and the principal investigator deems the event to be chronic or the participant to be stable. Research Nurses/Midwives will ask patients for any details of adverse events at five time-points: post procedure (if randomised to receive ES), at the participants' pregnancy test, and then, if pregnancy has been achieved, at 3 and 6 months post egg collection and finally 6 weeks post-partum.

AEs/SAEs will be collected up to the participants' final study related follow-up event. If embryo transfer does not occur, the Research Nurse/Midwife will contact the participant approximately 2 weeks after egg collection to identify if any adverse events have occurred. In the case of a negative pregnancy test, the site research team should make every effort to obtain AE data from the patient or the medical notes at routine clinical care contacts; no further contact will be made outside of routine clinical care.

Expected AEs will be those which occur regularly due to pregnancy, and expected SAEs are those events which are expected in the patient population as a result of the routine care/treatment of a patient. Expected SAEs and all AEs will be collected as part of the trial and entered into the eCRF, but will not be reported to regulatory bodies (NHS REC/sponsor). Unexpected SAEs will be reported to the Sheffield CTRU as soon as staff at the fertility unit becomes aware of the event.

For peer review only - http://bmjopen.bmj.com/site/about/guidelines.xhtml

All SAEs will be reviewed by the Data Monitoring & Ethics Committee (DMEC) and Trial Management Group (TMG) at regular intervals. The Chief Investigator (CI) will inform all Principal Investigators (PIs) concerned of relevant information that would adversely affect the safety of the participants.

Outcomes

Primary clinical outcome

- Live birth rate; based on the number of live births after 24 weeks gestation within the 10.5 month post egg collection follow-up period.

Secondary outcomes

- Acceptability and pain rating of the Endometrial Scratch procedure, a visual pain scale (likert) to assess their pain and tolerability assessment of the procedure within 30minutes of the initial ES procedure, 1 day later and then again 7 days after the ES.
- Implantation rate based on a positive serum Beta hCG on approximately day 14 following the egg collection or by a positive urine pregnancy test.
- Clinical pregnancy rate; an observation of viable intrauterine pregnancy with a positive heart pulsation seen on ultrasound at/after 8 weeks gestation
- Miscarriage rate as measured by spontaneous pregnancy loss (including pregnancy of unknown location (PUL) prior to 24 weeks gestation within the 10.5 month post egg collection follow-up period
- Ectopic pregnancy as measured by the rate of pregnancy outside the normal uterine cavity
- Multiple birth rate, defined as the birth of more than one living foetus after completed 24 weeks gestation
- Preterm delivery rate as measured by live birth after 24 weeks before 37 weeks gestation within the 10.5 month post egg collection follow-up period.
- Still birth rate based on the delivery of a still born foetus showing no signs of life after 24 weeks gestation within the 10.5 month post egg collection follow-up period.
- Details of participant's IVF cycles including number of eggs retrieved, number of embryos generated 1 day after egg collection, quality of the embryos transferred (using NEQAS grading) and the number of embryos replaced and day of embryo replacement.
- Adverse events
- Health resource use of the participant & patient costs

The trial includes a health economic component to assess the cost of the intervention per extra live birth from an NHS and social care perspective. Resource use will include the intervention costs for ES, the cost of IVF treatment, visits to the assisted conception unit and for those who conceive antenatal and post-natal visits, delivery costs and any hospital stays not related to birth for both mother and baby. The resource use questionnaire will collect

For peer review only - http://bmjopen.bmj.com/site/about/guidelines.xhtml

For peer review only

information on contacts with midwife and GP visits. A Patient Cost questionnaire will collect time taken to travel to appointments and loss of productivity. Unit costs will be derived from appropriate national sources and will include; NHS reference costs, Personal Social Service Research Unit costs and the **Office of National Statistics** [18–20]. The resource use questionnaire will be designed for this study and will draw on data collection tools developed in The School of Health and Related Research (ScHARR) and those collated by the Database for Instruments for Resource Use Measurement (DIRUM).

Blinding

Due to the nature of the intervention, it will not be possible to blind patients or clinicians to treatment allocation and since this trial evaluates objectively measured outcomes (pregnancy rates) that are unlikely to be affected by a placebo effect, it is not necessary to perform a sham procedure for the control group. The study statistician, TSC and health economist will be blinded to the allocation.

Trial monitoring and oversight committees

The trial will be overseen by the TSC and the DMEC, membership of both will consist of independent experts in the field. The TSC will include a patient representative. Both committees will review recruitment, study progress and adverse events. The DMEC will receive monthly reports of recruitment and adverse events and, at their meetings, will also consider emerging evidence from other trials or research on ES. They may advise the chair of the TSC at any time if, in their view, the trial should be stopped for ethical reasons, including concerns about patient safety.

Day-to-day running of the trial will be coordinated by the Trial Management Group (TMG), consisting of the grant co-applicants, plus members of the Jessop Wing Fertility Unit, Sheffield CTRU and patient representatives.

Statistical analysis

Primary analysis will be performed on the intention to treat population (all participants randomised into the trial). All statistical exploratory tests will be two-tailed at 5% nominal level. Baseline demographic (e.g. age), physical measurements (e.g. BMI), and health-related data will be described and summarised overall and for both treatment groups. The women, not the IVF cycle will be the unit of analysis. If the woman fails to get pregnant or does not have IVF treatment, they will be included in the analysis of the primary outcome as a negative outcome (i.e. non-live birth). For sensitivity analyses, per protocol (PP) analyses will also be undertaken which will be defined as for Endometrial Scratch participants in the intervention group, receiving the ES procedure as documented in the study protocol and undergoing IVF/ICSI in the subsequent menstrual cycle, including embryo transfer. For the control group, the PP population will receive IVF/ICSI including embryo transfer. Sub-group analyses will be undertaken to explore the effect of important variables related to the

For peer review only - http://bmjopen.bmj.com/site/about/guidelines.xhtml

participant and their treatment on the primary and secondary outcomes. These subgroups are:

- Day of embryo transfer (day 2, 3, 4, 5 or 6),
- Fertilisation method (IVF, IVF or ICSI, ICSI [spilt]),
- Type of protocol (long or antagonistic),
- Embryo transfer (single or double) and whether the embryo was fresh or frozen
- Previous history of consecutive miscarriages (0-2 vs >=3)

AEs will be reported as a proportion of all women randomised. Adverse events including serious adverse events will be compared between the two groups using a Fisher's Exact test, Chi-squared test or negative binomial regression model in case of repeated events per woman (as appropriate). A 95% CI for the difference in adverse event rate between the groups will also be calculated with associated point estimate depending on the method used.

Health economic results will be presented in the net-benefit framework and will allow for uncertainty using bootstrapping and probabilistic sensitivity analysis.

Ethics and dissemination

The study is registered on the ISRCTN database (reference 23800982) and has been approved by the South Berkshire Research Ethics Committee (reference 16/SC/0151). The findings of this trial will be submitted to peer- reviewed journals and abstracts to national and international conferences. Other stakeholder specific outputs in relevant formats will also be produced for commissioners, IVF practitioners, third sector and user advocacy organisations. A website will be established to promote the work of the trial. All knowledge transfer activity including translation will be informed by input from trial collaborators, the TSC and TMG to ensure the study is meeting the needs of the commissioners and audience.

Discussion

This trial will determine whether performing an ES procedure prior to 1st time IVF/ICSI treatment is an inexpensive, safe and well tolerated procedure that increases the live birth rate in women having SET. If shown to be the case, this will have a significant improvement in first cycle IVF success rates and potentially lead to significant cost savings to the NHS as fewer women would need to have repeat treatment cycles. This is particularly important in the current economic climate and with restrictions on funding and service provision. This will also have a significant impact for women, for whom the burden of repeated cycles is large.

For peer review only - http://bmjopen.bmj.com/site/about/guidelines.xhtml

Acknowledgements

We thank Dr Munya Dimairo - Research Fellow in Medical Statistics at the University of Sheffield for his contribution to the design/content of the protocol, The Jessop Wing Reproductive Health Public Advisory Panel as well as each local PI, Research Nurse/Midwife and research delivery team in the recruitment and management of patients.

Authors Contributions

Contributors: MM, CP, JC, KB, YC, SL, LM, JS, SW &, TY conceived the study, and contributed to study design, sample size calculations and analytical plans.

MM, CP, RC, JC, KB, YC, SL, LM, JS, SW & TY initiated the project, have assisted in developing the protocol and helped with implementation.

CP, RC, JC, & MM drafted the manuscript. All authors read and approved the final manuscript.

Competing interests

None declared.

Funding statement

This trial was funded by the NIHR Health Technology Assessment program grant number [14/08/45]

This article presents independent research supported by the National Institute for Health Research (NIHR). The views expressed are those of the author(s) and not necessarily those of the NHS, the NIHR or the Department of Health

References

1 Barash A, Dekel N, Fieldust S, *et al.* Local injury to the endometrium doubles the incidence of successful pregnancies in patients undergoing in vitro fertilization. *Fertil Steril* 2003;**79**:1317–22. doi:10.1016/S0015-0282(03)00345-5

2 El-Toukhy T, Sunkara S, Khalaf Y. Local endometrial injury and IVF outcome: A systematic review and meta-analysis. *Reprod Biomed Online* 2012;**25**:345–54. doi:10.1016/j.rbmo.2012.06.012

3 Raziel A, Schachter M, Strassburger D, *et al.* Favorable influence of local injury to the endometrium in intracytoplasmic sperm injection patients with high-order implantation failure. *Fertil Steril* 2007;**87**:198–201. doi:10.1016/j.fertnstert.2006.05.062

4 Huang SY, Wang C-J, Soong Y-K, *et al.* Site-specific endometrial injury improves implantation and pregnancy in patients with repeated implantation failures. *Reprod Biol Endocrinol* 2011;**9**:140. doi:10.1186/1477-7827-9-140

5 Simón C, Bellver J. Scratching beneath 'the Scratching Case': Systematic reviews and

For peer review only - http://bmjopen.bmj.com/site/about/guidelines.xhtml

metaanalyses, the back door for evidencebased medicine. Hum. Reprod. 2014;**29**:1618–21. doi:10.1093/humrep/deu126

6 Santamaria X, Katzorke N, Simón C. Endometrial ‘scratching’: What the data show. Curr. Opin. Obstet. Gynecol. 2016;**28**:242–9. doi:10.1097/GCO.0000000000000279

7 Potdar N, Gelbaya T, Nardo LG. Endometrial injury to overcome recurrent embryo implantation failure: a systematic review and meta-analysis. *Reprod Biomed Online* 2012;**25**:561–71. doi:10.1016/j.rbmo.2012.08.005

8 Nastri CO, Lensen SF, Gibreel A, *et al.* Endometrial injury in women undergoing assisted reproductive techniques. *Cochrane database Syst Rev* 2015;**3**:CD009517. doi:10.1002/14651858.CD009517.pub3

9 Karimzade MA, Oskouian H, Ahmadi S, *et al.* Local injury to the endometrium on the day of oocyte retrieval has a negative impact on implantation in assisted reproductive cycles: A randomized controlled trial. *Arch Gynecol Obstet* 2010;**281**:499–503. doi:10.1007/s00404-009-1166-1

10 Spencer EA, Mahtani KR, Goldacre B, *et al.* Claims for fertility interventions: a systematic assessment of statements on UK fertility centre websites. *BMJ Open* 2016;**6**:e013940. doi:10.1136/bmjopen-2016-013940

11 Lensen S, Sadler L, Farquhar C. Endometrial scratching for subfertility: Everyone’s doing it. *Hum Reprod* 2016;**31**:1241–4. doi:10.1093/humrep/dew053

12 van Hoogenhuijze NE, Torrance HL, Mol F, *et al.* Endometrial scratching in women with implantation failure after a first IVF/ICSI cycle; does it lead to a higher live birth rate? The SCRaTCH study: A randomized controlled trial (NTR 5342). *BMC Womens Health* 2017;**17**. doi:10.1186/s12905-017-0378-y

13 Lensen S, Martins W, Nastri C, *et al.* Pipelle for Pregnancy (PIP): study protocols for three randomised controlled trials. *Trials* 2016;**17**:216. doi:10.1186/s13063-016-1301-9

14 Human Fertilisation & Embryology Authority. Improving outcomes for fertility patients: Multiple births. A statistical report. 2011.

15 Ledger WL, Anumba D, Marlow N, *et al.* The costs to the NHS of multiple births after IVF treatment in the UK. *BJOG* 2006;**113**:21–5. doi:10.1111/j.1471-0528.2005.00790.x

16 Kalma Y, Granot I, Gnainsky Y, *et al.* Endometrial biopsy-induced gene modulation: first evidence for the expression of bladder-transmembranal uroplakin Ib in human endometrium. *Fertil Steril* 2009;**91**:1042–9, 1049-9. doi:10.1016/j.fertnstert.2008.01.043

17 Nastri CO, Gibreel A, Raine-Fenning N, *et al.* Endometrial injury in women undergoing assisted reproductive techniques. *Cochrane database Syst Rev* 2012;**7**:CD009517. doi:10.1002/14651858.CD009517.pub2

18 NHS reference costs 2012/13. 2013.

For peer review only - http://bmjopen.bmj.com/site/about/guidelines.xhtml

19 Curtis L. Unit costs of health and social care 2013. 2013.

20 Annual survey for hours and earnings (ASHE). 2012. http://www.ons.gov.uk/ons/rel/ashe/annual-survey-of-hours-and-earnings/2012-revised-results/index.html (accessed 12 Sep 2014).

For peer review only

For peer review only - http://bmjopen.bmj.com/site/about/guidelines.xhtml

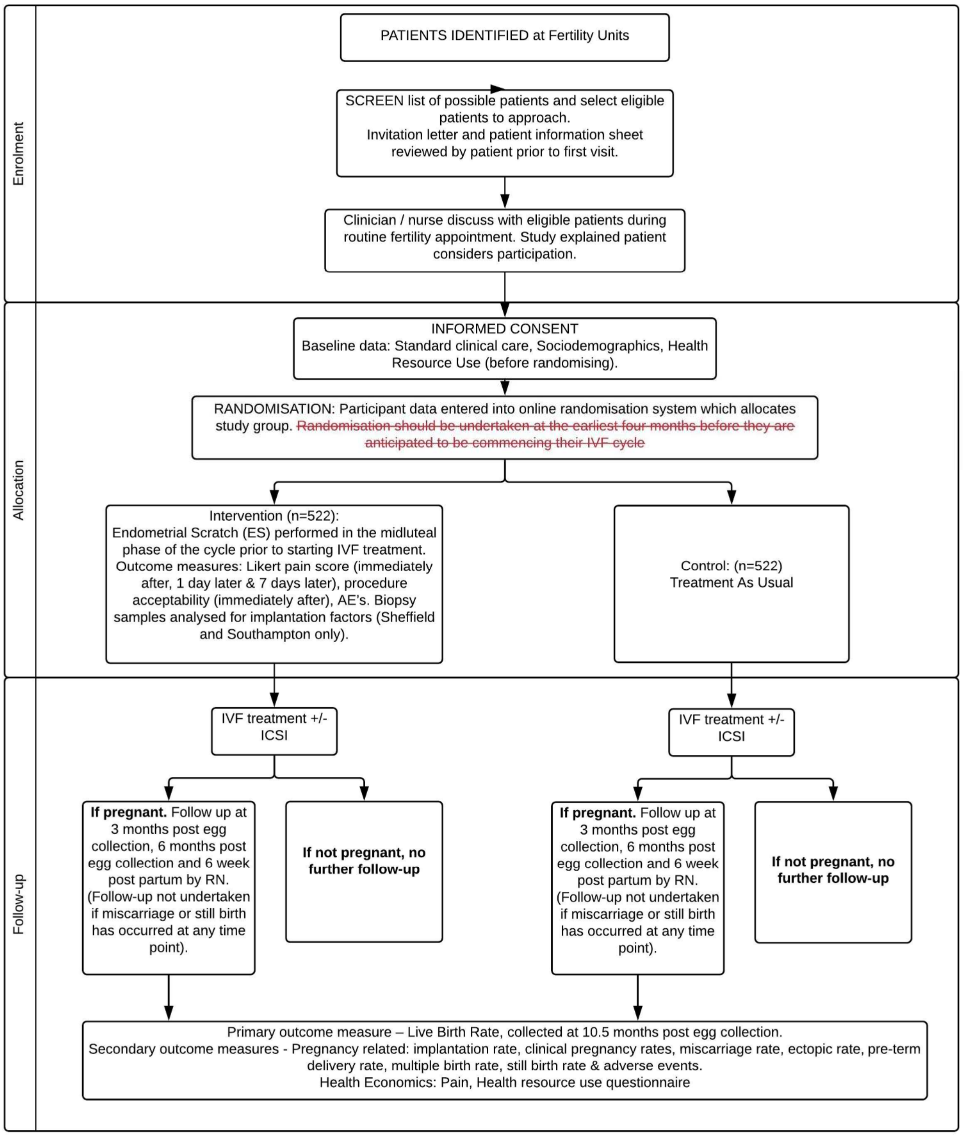

203x241mm (300 x 300 DPI)

BMJ Open

Induced Endometrial Trauma (endometrial scratch) in the mid-luteal menstrual cycle phase preceding first cycle IVF/ICSI versus usual IVF/ICSI therapy: Study protocol for a randomised controlled trial.

Journal:	*BMJ Open*
Manuscript ID	bmjopen-2017-020755.R2
Article Type:	Protocol
Date Submitted by the Author:	12-Mar-2018
Complete List of Authors:	Pye, Clare; Sheffield Teaching Hospitals NHS Foundation Trust, Obstetrics, Gynaecology & Neonatology<br>Chatters, Robin; University of Sheffield, Health and Related Research<br>Cohen, Judith; The University of Sheffield, School of Health and Related Research (ScHARR)<br>Brian, Kate; Fertility UK<br>Cheong, Ying; University of Southampton, Obstetrics and Gynaecology<br>Laird, Susan; Sheffield Hallam University<br>Mohiyiddeen, Lamiya; Central Manchester University Hospitals NHS Foundation Trust, Department Of Reproductive Medicine<br>Skull, Jonathan; Jessop Wing Hospital<br>Walters, Stephen; University of Sheffield, ScHARR<br>Young, T; The University of Sheffield, ScHARR<br>Metwally, Mostafa; Sheffield Teaching Hospitals NHS Foundation Trust, Obstetrics, Gynaecology & Neonatology
**Primary Subject Heading**:	Reproductive medicine
Secondary Subject Heading:	Obstetrics and gynaecology
Keywords:	Endometrial Trauma, Endometrial Scratch, IVF, Assisted Conception

SCHOLARONE™
Manuscripts

Title

Induced Endometrial Trauma (endometrial scratch) in the mid-luteal menstrual cycle phase preceding first cycle IVF/ICSI versus usual IVF/ICSI therapy:
Study protocol for a randomised controlled trial.

Author (*and corresponding author)*:

Clare Pye- RN
Lead Research Nurse/Clinical Trial Co-ordinator – Endometrial Scratch Trial
Rm 32, level 4. Jessop Wing
Sheffield Teaching hospitals NHS Foundation Trust
Tree Root Walk,
Sheffield. S10 2SF
Tel: 0114 2268194
E-mail: clare.pye@sth.nhs.uk

Co-Authors

Robin Chatters- Trial Manager
University of Sheffield- Clinical Trials Research Unit
Sheffield-UK

Dr Judith Cohen - Research Fellow/ Assistant Director CTRU - Design, Trials and Statistics (DTS). Research Fellow - The NIHR Research Design Service for Yorkshire & the Humber.
School of Health and Related Research (ScHARR)
University of Sheffield
Sheffield.
UK

Ms Kate Brian - Patient & Public Involvement (PPI)
Fertility Network Uk
London.
UK

Professor Ying Cheong - Professor of Reproductive Medicine, Honorary Consultant in Reproductive Medicine and Surgery.
Complete Fertility Centre Southampton
University Hospital Southampton NHS Foundation Trust.
Southampton.
UK

For peer review only

For peer review only - http://bmjopen.bmj.com/site/about/guidelines.xhtml

Dr Susan Laird – Head of the Department of Biosciences and Chemistry
Biomedical Research Centre
Sheffield Hallam University
Sheffield.
UK

Dr Lamiya Mohiyiddeen - Consultant Gynaecologist and Subspecialist in Reproductive Medicine and Surgery
Saint Mary's Hospital - Central Manchester University Hospitals
Manchester.
UK

Mr Jonathan Skull – Consultant in Reproductive Medicine
Fertility Unit - Jessop Wing
Sheffield Teaching Hospitals NHS Foundation Trust
Sheffield.
UK

Professor Stephen Walters - Trial Statistician
School of Health and Related Research and
NIHR Research Design Service Yorkshire and the Humber
University of Sheffield
Sheffield.
UK

Dr Tracey Young - Senior Researcher in Health Economics
Senior Researcher in Health Economics
Health Economics & Design Science
(ScHARR)
University of Sheffield
Sheffield.
UK

Mr Mostafa Metwally MD FRCOG - Consultant Gynaecologist and Subspecialist in Reproductive Medicine and Surgery
Fertility Unit - Jessop Wing
Sheffield Teaching Hospitals NHS Foundation Trust
Sheffield.
UK

For peer review only - http://bmjopen.bmj.com/site/about/guidelines.xhtml
For peer review only

Key words:

Endometrial Scratch

In Vitro Fertilisation

Endometrial Trauma

Assisted Conception

Randomised Controlled Trial

Abstract

Introduction

Endometrial Trauma commonly known as Endometrial Scratch (ES) has been shown to improve pregnancy rates in women with a history of repeated implantation failure undergoing In Vitro Fertilisation (IVF), with or without Intracytoplasmic Sperm Injection (ICSI). However, the procedure has not yet been fully explored in women having IVF/ICSI for the first time. This study aims to examine the effect of performing an ES in the mid-luteal phase prior to first time IVF/ICSI cycle on the chances of achieving a clinical pregnancy and live birth. If ES can influence this success rate there would be a significant cost saving to the NHS through decreasing the number of IVF/ICSI cycles necessary to achieve a pregnancy, increase the practice of single embryo transfer (SET) and consequently have a large impact on risks and costs associated with multiple pregnancies.

Methods & Analysis

This 30 month, UK, multi-centre, parallel group, RCT includes a 9month internal pilot and health economic analysis recruiting 1044 women from 16 Fertility Units. It will follow up participants to identify if IVF/ICSI has been successful and live birth has occurred up to 6 weeks post-partum. Primary analysis will be on an intention to treat basis. A sub-study of endometrial samples obtained during the ES will assess the role of immune factors in embryo implantation. Main trial recruitment commenced January 2017 and is ongoing.

Participants randomised to the intervention group will receive the ES procedure in the mid luteal phase of the preceding cycle prior to first time IVF/ICSI treatment versus usual IVF/ICSI treatment in the control group, with 1:1 randomisation. The primary outcome is live birth rate (LBR) after completed 24 weeks gestation.

Ethics and dissemination

South Central – Berkshire NREC approved the protocol. Findings will be submitted to peer-reviewed journals and abstracts to relevant national and international conferences.

Trial Registration number: ISRCTN: 23800982. Protocol Date: Version 5 Dated 20/07/2017

For peer review only - http://bmjopen.bmj.com/site/about/guidelines.xhtml

Strengths and limitations of this study

- This is the largest multicentre, pragmatic randomised controlled trial to date which aims to assess the effectiveness and cost effectiveness of performing the ES procedure in women having IVF/ICSI for the first time
- It aims to determine whether performing an ES is an acceptable and well tolerated procedure.
- Due to the nature of the intervention it is not possible to blind study participants or clinicians.
- Potential difficulty with recruitment if patients are not in equipoise about effectiveness of the ES procedure in first time IVF/ISCI cycles.

Background

The use of local endometrial trauma known as Endometrial Scratch (ES) to improve implantation rates in women undergoing assisted conception was first described in 2003 [1]. The procedure has since been explored in several studies mainly focusing on women with recurrent implantation failure and has been shown to significantly increase pregnancy rates by almost double [2–4]. However, uncertainty remains as to the therapeutic effect of ES, due to heterogeneity of the populations included - and the timing and exact protocol of ES used - in previous evaluations [5,6]. Three systematic reviews have summarised the evidence, however each included different studies [2,7,8]. A recent Cochrane review included fourteen randomised studies; seven in women with previous cycle failure, five in an unselected population and one in a first-time cycle [8]. The live birth rate meta-analysis combined trials regardless of the population (i.e. number of previous IVF cycles) and included five studies, reporting a risk ratio (RR) of 1.42 (1.08, 1.85), p=0.02. The odds of achieving a clinical pregnancy were also increased following ES with a RR of 1.34 (1.11, 1.62), p=0.002. The one trial conducted in women undergoing their first IVF cycle indicated the procedure was harmful with an OR of clinical pregnancy rate of 0.30 (0.14, 0.63) p=0.002 [9]. Notably, this trial performed the ES procedure at the time of oocyte retrieval and not in the month prior to the IVF cycle. Despite the concerns around the quality of evidence in using ES and that many of the trials undertaken so far have been small (most <150 participants), ES has been widely adopted into routine clinical practice in women with recurrent unsuccessful implantation and is currently being provided in some fertility units where women are having IVF/ICSI for the first time [10,11]. Two large trials are currently in progress to determine if ES is beneficial in women undergoing their 2nd IVF cycle [12] and a sample of women undergoing any IVF cycle [13]. Therefore, given the lack of evidence for the effectiveness of ES in women undergoing their 1st cycle of IVF/ICSI, it is essential that a large well controlled multi-centre trial is conducted to fully investigate the effectiveness and safety of this technique.

The Human Fertilisation and Embryology Authority (HFEA) state in their statistical report into multiple births that the risks associated with multiple births is the single biggest health risk associated with fertility treatment [14]. Multiple births carry risks to the health of both

For peer review only - http://bmjopen.bmj.com/site/about/guidelines.xhtml

the mother and the babies and that birth of a healthy singleton child, born at full term, is therefore the safest outcome of fertility treatment for both mother and child and is best achieved through promoting the practice of SET.

If ES can improve the implantation potential of the embryo and therefore improve success rates, ES may encourage an expansion of current SET policies. Inclusion of women with a lower chance of having cryopreserved embryos and a more general increase in the implementation of the practice of SET, could consequently have a large impact on the risks and costs associated with multiple pregnancies as a result of IVF [15].

The exact mechanism by which ES may improve implantation is not yet known, however it is known that implantation is a complex process involving a number of inflammatory mediators including uterine natural killer cells, leukaemia inhibitory factor and interleukin 15 [13]. It is possible that ES may lead to the activation of inflammatory cells such as macrophages and dendritic cells, and release of inflammatory mediators such as tumour necrosis factor-α, interleukin-15, growth-regulated oncogene-α and macrophage inflammatory protein 1B [14].

ES has also been shown to cause the modulation of several endometrial genes that may be involved in membrane stability during the process of implantation such as bladder transmembranal protein (UPIb) and adipose differentiation-related protein and mucin 1 [16].

ES is routinely performed as an outpatient procedure. Risks have been identified in a previous study when the procedure was undertaken on the day of oocyte retrieval (reduced implantation and pregnancy rates) [9]; however, the procedure is not known to be associated with any particular risks when undertaken in the menstrual cycle preceding that of IVF therapy, apart from period like discomfort whilst performing the procedure. Taking simple analgesics prior to the procedure usually alleviates this. As with any intrauterine procedure there is a potential for intrauterine infection. However women attending for fertility treatment are usually screened for serious vaginal infections such as chlamydia to minimise the risk of any spread of infection when performing the embryo transfer procedure, a similar procedure to an ES as it involves the insertion of a catheter into the uterine cavity.

The main objectives of this trial are to assess the clinical and cost effectiveness of the ES procedure in women aged between 18 and 37 years (inclusive) undergoing their first IVF/ICSI cycle using either antagonist or long protocols to see if it could potentially improve implantation rates and hence encourage the practice of single embryo replacement. A sub-study will be undertaken in two of the fertility units where endometrial samples obtained from the ES procedure will be stored for later analysis to identify endometrial factors associated with successful pregnancy outcome.

For peer review only - http://bmjopen.bmj.com/site/about/guidelines.xhtml

Method and Analysis

The Endometrial Scratch Trial is a multi-centre, parallel group, randomised controlled trial to examine the clinical, cost effectiveness and safety of an ES performed in the mid-luteal phase of the preceding cycle prior to a first time IVF/ICSI cycle. Eligible participants will be randomised to either the treatment as usual (TAU) arm, consisting of usual IVF treatment, or the intervention arm where ES will be performed followed by usual IVF treatment. The overall study design is illustrated below in the study flow chart (figure 1).

Patient and Public Involvement

The study was reviewed by couples waiting to commence IVF treatment and then by the members of The Jessop Wing Reproductive Health Public Advisory Panel (PPI) at the Jessop Wing-Sheffield. All were asked to provide input into the lay summary, recruitment strategy, visit schedule and benefits of the proposed study to the patient & the NHS. We asked about their experience of assisted conception, the things they liked and disliked, and the potential difficulties or barriers to attending for treatment, randomisation to the TAU arm and how this might affect recruitment but clarified that if the trial showed an increase in the scratch arm assisting embryo implantation, then it would form part of the routine care pathway in the future. A member of the panel agreed to become the Service User Representative, is a member of the Steering committee and attended the Trial Investigator/set-up meeting providing a patients view of all aspects associated with IVF. All PPI members have provided input into the patient facing documents on an ongoing basis and prior to submission to ethics and are aware of recruitment and the conduct of the study at ongoing PPI events held on a bimonthly basis within the Directorate.

The most significant changes to the HTA grant influenced by the PPI members were in relation to trial follow-up procedures as they felt only women who achieved a pregnancy should be followed up. They also wanted to ensure continuity across the participating centres when performing the follow-up visits and requested a proforma be designed to ensure all research nurses/midwives capture the same information.

Upon completion of the trial the results will be summarised in plain English and distributed to participants and patient support groups such as Infertility Network UK with the assistance of our service-user collaborators. We will promote the transfer of knowledge to wider audiences including the general public (e.g. including short, user-friendly articles/briefings in relevant newsletters, magazines and periodicals, user groups/forums).

Figure 1. Study flow chart.

The trial consists of two phases - an internal pilot to assess feasibility of recruitment and delivery of the intervention, and a two year main recruitment phase.

For peer review only - http://bmjopen.bmj.com/site/about/guidelines.xhtml

The trial will commence with a 9 month internal pilot recruitment phase across approximately 6 UK fertility units to justify whether or not the recruitment strategy and the scheduling of the endometrial scratch procedure are feasible and will use the same trial procedures as described for the main trial.

The trial is collaboration between research staff at The Jessop Wing, Sheffield Teaching Hospital NHS Foundation Trust & The University of Sheffield - Clinical Trials Research Unit (CTRU) who is responsible for the conduct of the trial. Funding to run the trial has been awarded by the National Institute of Healthy Research (NIHR) Health Technology Assessment (HTA). At the end of the pilot phase, the Trial Steering Committee (TSC) will report to the funder on whether the feasibility criteria have been met and whether the trial should continue.
The trial will be conducted in compliance with the approved protocol, GCP and regulatory requirements. Main trial recruitment commenced January 2017 and is ongoing.

Sheffield CTRU will aggregate feasibility of the research and intervention protocols based on the following outcomes.
The trial will be considered infeasible and will be stopped if either of the following conditions apply:

1. Feasibility of recruitment to the main trial: defined as recruitment of fewer than 108 participants (75% of the 144 target) during the internal pilot phase.

2. Scheduling of the ES procedure: defined as less than 75% of women scheduled to receive their ES procedure have received the ES at the correct time point.

Recruitment
Upon successful completion of the pilot the main trial will aim to recruit women from 16 UK Fertility Units requiring first time IVF treatment. Participation is entirely voluntary and choosing not to participate will not negatively influence the woman's treatment in any way. Furthermore consent can be withdrawn at any stage. Women who are about to undergo their first cycle of IVF/ICSI will be identified by screening patients referred for these treatments. Eligible women will be sent information regarding the trial in the post or via e-mail or may be alerted to the trial via the trial website or posters displayed at the fertility unit. If they are interested in participating they will be invited to discuss the trial with their fertility team at their next routine appointment.

Prior to randomisation full written informed consent will be obtained by a suitably trained Doctor or Research Nurse/Midwife at a clinic visit. The participant will complete a study specific resource use questionnaire prior to randomisation to collect health care usage in the previous 3 months; baseline data will be collected at this visit and participants will be randomly allocated to either the intervention or usual care arm of the trial.

For peer review only - http://bmjopen.bmj.com/site/about/guidelines.xhtml

Detailed methods of the Endometrial Scratch trial are described in the Endometrial Scratch protocol available on the website – https://www.sheffield.ac.uk/scratchtrial

Women will be included and considered suitable if they meet the following eligibility criteria:

Inclusion criteria

1. Women expected to be aged between 18 and 37 years (inclusive) at time of egg collection.
2. First time IVF with or without ICSI treatment using the antagonist or long protocol only.
3. Expected to receive treatment using fresh embryos.
4. Expected good responders to treatment, with:
    a. Ovulatory menstrual cycle (Regular menstrual cycles defined by clinical judgement or with ovulatory levels of mid-luteal serum progesterone as defined by local laboratory protocols)
    b. Normal uterine cavity (assessed by transvaginal sonography at screening and no endometrial abnormalities such as , suspected intrauterine adhesions, uterine septa, submucosal fibroids or intramural fibroids exceeding 4 cm in diameter as assessed by the investigator that would require treatment to facilitate pregnancy).
    c. Expected good ovarian reserve (assessed clinically, biochemically (FSH< 10 & normal follicular phase oestradiol levels and or normal AMH), and or sonographically (antral follicle counts) and no history of previous radiotherapy or chemotherapy). [All laboratory/ultrasound standards based on local normal reference ranges.]
    d. Single embryo transfer (SET) expected.
5. Local procedures have been / will be followed to exclude relevant vaginal/uterine infections prior to starting treatment.
6. Willing to use an appropriate method of barrier contraception if randomised to Endometrial Scratch (ES) in the cycle where the ES procedure is performed. .
7. Understands/willing to comply with the protocol.

Exlcusion criteria

1. Previous trauma/surgery to the endometrium (e.g. resection of submucous fibroid, intrauterine adhesions.).
2. BMI of 35 kg/m2 or greater.
3. Known grade 4 (severe) endometriosis.
4. Currently participating in any other fertility study involving medical/surgical intervention.
5. Expected to receive protocols other than antagonist or long (e.g. ultra-long protocol).
6. An endometrial scratch (or similar procedure, e.g. endometrial biopsy for the collection of Natural Killer Cells) is planned.
7. Previously randomised into this trial.

For peer review only - http://bmjopen.bmj.com/site/about/guidelines.xhtml

Sampling

The primary outcome is the LBR. This is defined as a live birth after completed 24 weeks gestation, within the 10.5 month post egg collection follow-up period.This time-period will enable the collection of any neonatal deaths (up to 6 weeks post-partum). The denominator for calculating the LBR will be the number of women randomised to each group. Data from the HFEA suggests a live birth rate of 32.8% in women under 35 and 27.3 % in women aged 35-37. The sample size calculation assumes a 30% LBR in the control group and that an absolute increase of 10%, to a 40% LBR (a relative risk of 1.33) in the intervention groups is of clinical and practical importance. The effect size, a 10% absolute difference in LBR, we are proposing is large but we believe an effect of such magnitude is needed to change clinical practice (there is a 5% absolute difference in LBR between women aged under 35 and 35-37) and is less than that observed in the systematic reviews described above (where, at the time of sample size calculation, the relative risk estimates for live birth ranged from 1.83 to 2.46) [2,17]. To have a 90% power of detecting this difference or more, in LBR rates between the groups, as statistically significant at the 5% two-sided level, will require 496 women per group (992 in total). Adjusting for a predicted drop-out rate of 5% (due to anticipated difficulties of follow-up for patients who have been referred from NHS Trusts other than the participating Fertility Unit) we will require 1044 participants.

Study procedures

Following randomisation women in the intervention arm will have the ES procedure performed in the mid-luteal phase of the preceding cycle prior to their planned IVF/ICSI cycle in the outpatient setting of the fertility unit. The choice of screening for infection prior to the procedure or the administration of antibiotics will be left to individual unit according to their local established protocols and procedures. Women can be randomised any time up until they start their IVF cycle, although it may be necessary for the participant to delay her IVF if randomised to the intervention arm. This decision to delay should be made and agreed by both the patient and her fertility team before randomisation is undertaken. The oral contraceptive pill can be used following randomisation for the purposes of cycle programming but women must be having ovulatory periods at the point of entry into the trial. Women randomised to TAU will continue with their IVF/ICSI as planned and will not receive the ES procedure.

Following delivery of the ES, participants will undergo IVF/ICSI in line with local procedures. Following successful embryo transfer (in both groups) a pregnancy test will be performed and adverse events will be collected. In cases where women do not undergo embryo transfer, every effort will be made by the research team to collect any adverse event information from either the patient or the medical notes. If a pregnancy is confirmed the woman is discharged to normal antenatal care as per standard practice. It is the intention to obtain the pregnancy status of all women once randomised including the outcome of all spontaneous pregnancies, the first frozen embryo transfer if no fresh transfer has been undertaken as well as those that delay treatment following the ES. Women will not be

For peer review only - http://bmjopen.bmj.com/site/about/guidelines.xhtml

followed-up if they withdraw their consent from the trial. Data will also be collected regarding participants who have received an ES outside the trial.

Randomisation

The randomisation schedule will be generated by Sheffield CTRU prior to the start of the trial; access to the schedule will be limited only to the trial statistician. The randomisation sequence will be computer generated and stratified by site and protocol (antagonist or long protocol). Random permuted blocks of variable size will be used to ensure enough participants are allocated evenly to each arm of the trial at each site. Research staff at recruiting centres will be unable to access the randomisation sequence and will use a web based computer system with restricted access rights to enter participant details; randomisation outcome will then be revealed. Re-randomisation will not be permitted.

Trial Intervention

ES is a minor procedure of 10 to 20 minute duration that will be performed in an outpatient setting at local IVF centres in line with local procedures and the trial standard operation procedure (SOP). The participant will be required to use a barrier method of contraception (if necessary) during the menstrual cycle in which the ES will be performed. During ES, a speculum is inserted into the vagina and the cervix exposed and cleaned. A pipelle or similar endometrial sampler is then inserted into the cavity of the uterus; negative pressure is applied by withdrawal of the plunger. The sampler is rotated and withdrawn several times so that tissue appears in the transparent tube. The sampler and speculum are then removed. If no tissue is seen in the transparent sampler, this is an indication that the sampler was not fully inside the uterine cavity and therefore the procedure is repeated. Following the procedure women will complete a visual pain scale (likert) to assess their pain and tolerability assessment of the procedure within 30 minutes of the initial ES and then again at 24 hours and 7 days post procedure via an automated text message.

Compliance to the intervention will be ascertained through the clinician or Research Nurse/Midwife recording whether or not the patient has a) attended the clinic for the ES procedure and b) received the ES procedure as per protocol. Any deviation from the protocol will be noted and reported as per the Sheffield CTRU SOP.

Follow-up

Patient follow up will continue until either the 1st cycle of IVF has been completed or the resulting pregnancy has concluded. If no pregnancy is confirmed the study is complete (regardless of which group the woman is randomised to). Pregnant women will be followed-up at 3 and 6 months post egg collection and then 6 weeks post-partum to collect pregnancy outcome and safety data. If the pregnancy is ongoing at 3 months and 6 week post-partum, a health resource use questionnaire will be sent to the patient for completion. If a spontaneous pregnancy is achieved between randomisation and IVF treatment, the pregnancy will be followed up as described above, instead of egg collection, the date of the

For peer review only - http://bmjopen.bmj.com/site/about/guidelines.xhtml

last menstrual period (LMP) will be used to schedule the 3/6 month & 6 week post-partum follow ups.

Safety considerations, safety monitoring and AE reporting

All Adverse Events (AEs) and Serious Adverse Events (SAEs) will be recorded by the local research team at each Fertility Unit. All AEs/SAEs will be followed up until satisfactory resolution or until the treating Clinician and the Principal Investigator deems the event to be chronic or the participant to be stable. Research Nurses/Midwives will ask patients for any details of adverse events at five time-points: post procedure (if randomised to receive ES), at the participants' pregnancy test, and then, if pregnancy has been achieved, at 3 and 6 months post egg collection and finally 6 weeks post-partum.

AEs/SAEs will be collected up to the participants' final study related follow-up event. If embryo transfer does not occur, the Research Nurse/Midwife will contact the participant approximately 2 weeks after egg collection to identify if any adverse events have occurred. In the case of a negative pregnancy test, the site research team should make every effort to obtain AE data from the patient or the medical notes at routine clinical care contacts; no further contact will be made outside of routine clinical care.

Expected AEs will be those which occur regularly due to pregnancy, and expected SAEs are those events which are expected in the patient population as a result of the routine care/treatment of a patient. Expected SAEs and all AEs will be collected as part of the trial and entered into the eCRF, but will not be reported to regulatory bodies (NHS REC/sponsor). Unexpected SAEs will be reported to the Sheffield CTRU as soon as staff at the fertility units become aware of the event.

All SAEs will be reviewed by the Data Monitoring & Ethics Committee (DMEC) and Trial Management Group (TMG) at regular intervals. The Chief Investigator (CI) will inform all Principal Investigators (PIs) concerned of relevant information that would adversely affect the safety of the participants.

Outcomes

Primary clinical outcome

- Live birth rate; based on the number of live births after 24 weeks gestation within the 10.5 month post egg collection follow-up period.

Secondary outcomes

- Acceptability and pain rating of the Endometrial Scratch procedure, a visual pain scale (likert) to assess their pain and tolerability assessment of the procedure within 30minutes of the initial ES procedure, 1 day later and then again 7 days after the ES.
- Implantation rate based on a positive serum Beta hCG on approximately day 14 following the egg collection or by a positive urine pregnancy test.

For peer review only - http://bmjopen.bmj.com/site/about/guidelines.xhtml

- Clinical pregnancy rate; an observation of viable intrauterine pregnancy with a positive heart pulsation seen on ultrasound at/after 8 weeks gestation
- Miscarriage rate as measured by spontaneous pregnancy loss (including pregnancy of unknown location (PUL) prior to 24 weeks gestation within the 10.5 month post egg collection follow-up period
- Ectopic pregnancy as measured by the rate of pregnancy outside the normal uterine cavity
- Multiple birth rate, defined as the birth of more than one living foetus after completed 24 weeks gestation
- Preterm delivery rate as measured by live birth after 24 weeks before 37 weeks gestation within the 10.5 month post egg collection follow-up period.
- Still birth rate based on the delivery of a still born foetus showing no signs of life after 24 weeks gestation within the 10.5 month post egg collection follow-up period.
- Details of participant's IVF cycles including number of eggs retrieved, number of embryos generated 1 day after egg collection, quality of the embryos transferred (using NEQAS grading) and the number of embryos replaced and day of embryo replacement.
- Adverse events
- Health resource use of the participant & patient costs

The trial includes a health economic component to assess the cost of the intervention per extra live birth from an NHS and social care perspective. Resource use will include the intervention costs for ES, the cost of IVF treatment, visits to the Assisted Conception Unit and for those who conceive antenatal and post-natal visits, delivery costs and any hospital stays not related to birth for both mother and baby. The resource use questionnaire will collect information on contacts with midwife and GP visits. A Patient Cost questionnaire will collect time taken to travel to appointments and loss of productivity. Unit costs will be derived from appropriate national sources and will include; NHS reference costs, Personal Social Service Research Unit costs and the **Office of National Statistics** [18–20]. The resource use questionnaire will be designed for this study and will draw on data collection tools developed in The School of Health and Related Research (ScHARR) and those collated by the Database for Instruments for Resource Use Measurement (DIRUM).

Blinding

Since this trial evaluates objectively measured outcomes (pregnancy rates) that are unlikely to be affected by a placebo effect, participants will not be blinded to treatment allocation; it is therefore not necessary to perform a sham procedure for the control group. The study statistician, TSC and health economist will be blinded to the allocation.

Trial monitoring and oversight committees

The trial will be overseen by the TSC and the DMEC, membership of both will consist of independent experts in the field. The TSC will include a patient representative. Both

For peer review only - http://bmjopen.bmj.com/site/about/guidelines.xhtml

committees will review recruitment, study progress and adverse events. The DMEC will receive monthly reports of recruitment and adverse events and, at their meetings, will also consider emerging evidence from other trials or research on ES. They may advise the chair of the TSC at any time if, in their view, the trial should be stopped for ethical reasons, including concerns about patient safety.

Day-to-day running of the trial will be coordinated by the Trial Management Group (TMG), consisting of the grant co-applicants, plus members of the Jessop Wing Fertility Unit, Sheffield CTRU and Patient representatives.

Statistical analysis

Primary analysis will be performed on the intention to treat population (all participants randomised into the trial). All statistical exploratory tests will be two-tailed at 5% nominal level. Baseline demographic (e.g. age), physical measurements (e.g. BMI), and health-related data will be described and summarised overall and for both treatment groups. The women, not the IVF cycle will be the unit of analysis. If the woman fails to get pregnant or does not have IVF treatment, they will be included in the analysis of the primary outcome as a negative outcome (i.e. non-live birth). For sensitivity analyses, per protocol (PP) analyses will also be undertaken which will be defined as for Endometrial Scratch participants in the intervention group, receiving the ES procedure as documented in the study protocol and undergoing IVF/ICSI in the subsequent menstrual cycle, including embryo transfer. For the control group, the PP population will receive IVF/ICSI including embryo transfer. Sub-group analyses will be undertaken to explore the effect of important variables related to the participant and their treatment on the primary and secondary outcomes. These subgroups are:

- Day of embryo transfer (day 2, 3, 4, 5 or 6),
- Fertilisation method (IVF, IVF or ICSI, ICSI [spilt]),
- Type of protocol (long or antagonistic),
- Embryo transfer (single or double) and whether the embryo was fresh or frozen
- Previous history of consecutive miscarriages (0-2 vs >=3)

AEs will be reported as a proportion of all women randomised. Adverse events including serious adverse events will be compared between the two groups using a Fisher's Exact test, Chi-squared test or negative binomial regression model in case of repeated events per woman (as appropriate). A 95% CI for the difference in adverse event rate between the groups will also be calculated with associated point estimate depending on the method used.

Health economic results will be presented in the net-benefit framework and will allow for uncertainty using bootstrapping and probabilistic sensitivity analysis.

For peer review only - http://bmjopen.bmj.com/site/about/guidelines.xhtml

Ethics and dissemination

The study is registered on the ISRCTN database (reference 23800982) and has been approved by the South Berkshire Research Ethics Committee (reference 16/SC/0151). The findings of this trial will be submitted to peer- reviewed journals and abstracts to national and international conferences. Other stakeholder specific outputs in relevant formats will also be produced for commissioners, IVF practitioners, third sector and user advocacy organisations. A website will be established to promote the work of the trial. All knowledge transfer activity including translation will be informed by input from trial collaborators, the TSC and TMG to ensure the study is meeting the needs of the commissioners and audience.

Discussion

This trial will determine whether performing an ES procedure prior to 1st time IVF/ICSI treatment is an inexpensive, safe and well tolerated procedure that increases the live birth rate in women having SET. If shown to be the case, this will have a significant improvement in first cycle IVF success rates and potentially lead to significant cost savings to the NHS as fewer women would need to have repeat treatment cycles. This is particularly important in the current economic climate and with restrictions on funding and service provision. This will also have a significant impact for women, for whom the burden of repeated cycles is large.

Acknowledgements

We thank Dr Munya Dimairo - Research Fellow in Medical Statistics at the University of Sheffield for his contribution to the design/content of the protocol, The Jessop Wing Reproductive Health Public Advisory Panel as well as each local PI, Research Nurse/Midwife and research delivery team in the recruitment and management of patients.

Authors Contributions

Contributors: MM, CP, JC, KB, YC, SL, LM, JS, SW &, TY conceived the study, and contributed to study design, sample size calculations and analytical plans.

MM, CP, RC, JC, KB, YC, SL, LM, JS, SW & TY initiated the project, have assisted in developing the protocol and helped with implementation.

CP, RC, JC, & MM drafted the manuscript. All authors read and approved the final manuscript.

Competing interests

None declared.

Funding statement

This trial was funded by the NIHR Health Technology Assessment program grant number [14/08/45]

For peer review only - http://bmjopen.bmj.com/site/about/guidelines.xhtml

This article presents independent research supported by the National Institute for Health Research (NIHR). The views expressed are those of the author(s) and not necessarily those of the NHS, the NIHR or the Department of Health

References

1 Barash A, Dekel N, Fieldust S, *et al.* Local injury to the endometrium doubles the incidence of successful pregnancies in patients undergoing in vitro fertilization. *Fertil Steril* 2003;**79**:1317–22. doi:10.1016/S0015-0282(03)00345-5

2 El-Toukhy T, Sunkara S, Khalaf Y. Local endometrial injury and IVF outcome: A systematic review and meta-analysis. *Reprod Biomed Online* 2012;**25**:345–54. doi:10.1016/j.rbmo.2012.06.012

3 Raziel A, Schachter M, Strassburger D, *et al.* Favorable influence of local injury to the endometrium in intracytoplasmic sperm injection patients with high-order implantation failure. *Fertil Steril* 2007;**87**:198–201. doi:10.1016/j.fertnstert.2006.05.062

4 Huang SY, Wang C-J, Soong Y-K, *et al.* Site-specific endometrial injury improves implantation and pregnancy in patients with repeated implantation failures. *Reprod Biol Endocrinol* 2011;**9**:140. doi:10.1186/1477-7827-9-140

5 Simón C, Bellver J. Scratching beneath 'the Scratching Case': Systematic reviews and metaanalyses, the back door for evidencebased medicine. Hum. Reprod. 2014;**29**:1618–21. doi:10.1093/humrep/deu126

6 Santamaria X, Katzorke N, Simón C. Endometrial 'scratching': What the data show. Curr. Opin. Obstet. Gynecol. 2016;**28**:242–9. doi:10.1097/GCO.0000000000000279

7 Potdar N, Gelbaya T, Nardo LG. Endometrial injury to overcome recurrent embryo implantation failure: a systematic review and meta-analysis. *Reprod Biomed Online* 2012;**25**:561–71. doi:10.1016/j.rbmo.2012.08.005

8 Nastri CO, Lensen SF, Gibreel A, *et al.* Endometrial injury in women undergoing assisted reproductive techniques. *Cochrane database Syst Rev* 2015;**3**:CD009517. doi:10.1002/14651858.CD009517.pub3

9 Karimzade MA, Oskouian H, Ahmadi S, *et al.* Local injury to the endometrium on the day of oocyte retrieval has a negative impact on implantation in assisted reproductive cycles: A randomized controlled trial. *Arch Gynecol Obstet* 2010;**281**:499–503. doi:10.1007/s00404-009-1166-1

10 Spencer EA, Mahtani KR, Goldacre B, *et al.* Claims for fertility interventions: a systematic assessment of statements on UK fertility centre websites. *BMJ Open* 2016;**6**:e013940. doi:10.1136/bmjopen-2016-013940

11 Lensen S, Sadler L, Farquhar C. Endometrial scratching for subfertility: Everyone's doing it. *Hum Reprod* 2016;**31**:1241–4. doi:10.1093/humrep/dew053

For peer review only - http://bmjopen.bmj.com/site/about/guidelines.xhtml

12 van Hoogenhuijze NE, Torrance HL, Mol F, *et al.* Endometrial scratching in women with implantation failure after a first IVF/ICSI cycle; does it lead to a higher live birth rate? The SCRaTCH study: A randomized controlled trial (NTR 5342). *BMC Womens Health* 2017;**17**. doi:10.1186/s12905-017-0378-y

13 Lensen S, Martins W, Nastri C, *et al.* Pipelle for Pregnancy (PIP): study protocols for three randomised controlled trials. *Trials* 2016;**17**:216. doi:10.1186/s13063-016-1301-9

14 Human Fertilisation & Embryology Authority. Improving outcomes for fertility patients: Multiple births. A statistical report. 2011.

15 Ledger WL, Anumba D, Marlow N, *et al.* The costs to the NHS of multiple births after IVF treatment in the UK. *BJOG* 2006;**113**:21–5. doi:10.1111/j.1471-0528.2005.00790.x

16 Kalma Y, Granot I, Gnainsky Y, *et al.* Endometrial biopsy-induced gene modulation: first evidence for the expression of bladder-transmembranal uroplakin Ib in human endometrium. *Fertil Steril* 2009;**91**:1042–9, 1049-9. doi:10.1016/j.fertnstert.2008.01.043

17 Nastri CO, Gibreel A, Raine-Fenning N, *et al.* Endometrial injury in women undergoing assisted reproductive techniques. *Cochrane database Syst Rev* 2012;**7**:CD009517. doi:10.1002/14651858.CD009517.pub2

18 NHS reference costs 2012/13. 2013.

19 Curtis L. Unit costs of health and social care 2013. 2013.

20 Annual survey for hours and earnings (ASHE). 2012. http://www.ons.gov.uk/ons/rel/ashe/annual-survey-of-hours-and-earnings/2012-revised-results/index.html (accessed 12 Sep 2014).

For peer review only - http://bmjopen.bmj.com/site/about/guidelines.xhtml

For peer review only

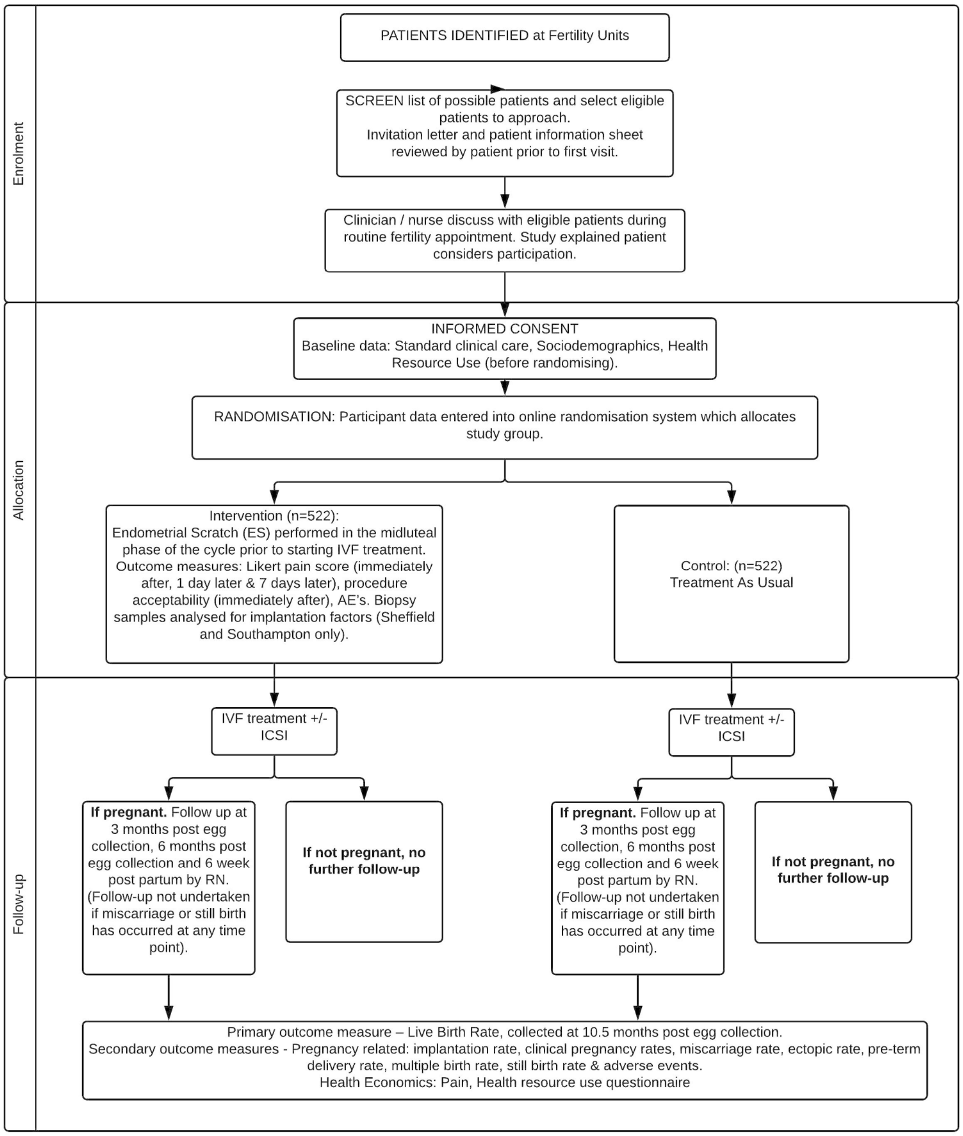

203x241mm (300 x 300 DPI)

Induced Endometrial Trauma (endometrial scratch) in the mid-luteal menstrual cycle phase preceding first cycle IVF/ICSI versus usual IVF/ICSI therapy: Study protocol for a randomised controlled trial.

Journal:	*BMJ Open*
Manuscript ID	bmjopen-2017-020755.R3
Article Type:	Protocol
Date Submitted by the Author:	29-Mar-2018

Complete List of Authors:	Pye, Clare; Sheffield Teaching Hospitals NHS Foundation Trust, Obstetrics, Gynaecology & Neonatology<br>Chatters, Robin; University of Sheffield, Health and Related Research<br>Cohen, Judith; The University of Sheffield, School of Health and Related Research (ScHARR)<br>Brian, Kate; Fertility UK<br>Cheong, Ying; University of Southampton, Obstetrics and Gynaecology<br>Laird, Susan; Sheffield Hallam University<br>Mohiyiddeen, Lamiya; Central Manchester University Hospitals NHS Foundation Trust, Department Of Reproductive Medicine<br>Skull, Jonathan; Jessop Wing Hospital<br>Walters, Stephen; University of Sheffield, ScHARR<br>Young, T; The University of Sheffield, ScHARR<br>Metwally, Mostafa; Sheffield Teaching Hospitals NHS Foundation Trust, Obstetrics, Gynaecology & Neonatology

**Primary Subject Heading**:	Reproductive medicine
Secondary Subject Heading:	Obstetrics and gynaecology
Keywords:	Endometrial Trauma, Endometrial Scratch, IVF, Assisted Conception

SCHOLARONE™
Manuscripts

Title

Induced Endometrial Trauma (endometrial scratch) in the mid-luteal menstrual cycle phase preceding first cycle IVF/ICSI versus usual IVF/ICSI therapy:
Study protocol for a randomised controlled trial.

Author (*and corresponding author)*:
Clare Pye- RN
Lead Research Nurse/Clinical Trial Co-ordinator – Endometrial Scratch Trial
Rm 32, level 4. Jessop Wing
Sheffield Teaching hospitals NHS Foundation Trust
Tree Root Walk,
Sheffield. S10 2SF
Tel: 0114 2268194
E-mail: clare.pye@sth.nhs.uk

Co-Authors

Robin Chatters- Trial Manager
University of Sheffield- Clinical Trials Research Unit
Sheffield-UK

Dr Judith Cohen - Research Fellow/ Assistant Director CTRU - Design, Trials and Statistics (DTS). Research Fellow - The NIHR Research Design Service for Yorkshire & the Humber.
School of Health and Related Research (ScHARR)
University of Sheffield
Sheffield.
UK

Ms Kate Brian - Patient & Public Involvement (PPI)
Fertility Network Uk
London.
UK

Professor Ying Cheong - Professor of Reproductive Medicine, Honorary Consultant in Reproductive Medicine and Surgery.
Complete Fertility Centre Southampton
University Hospital Southampton NHS Foundation Trust.
Southampton.
UK

For peer review only - http://bmjopen.bmj.com/site/about/guidelines.xhtml

Dr Susan Laird – Head of the Department of Biosciences and Chemistry
Biomedical Research Centre
Sheffield Hallam University
Sheffield.
UK

Dr Lamiya Mohiyiddeen - Consultant Gynaecologist and Subspecialist in Reproductive Medicine and Surgery
Saint Mary's Hospital - Central Manchester University Hospitals
Manchester.
UK

Mr Jonathan Skull – Consultant in Reproductive Medicine
Fertility Unit - Jessop Wing
Sheffield Teaching Hospitals NHS Foundation Trust
Sheffield.
UK

Professor Stephen Walters - Trial Statistician
School of Health and Related Research and
NIHR Research Design Service Yorkshire and the Humber
University of Sheffield
Sheffield.
UK

Dr Tracey Young - Senior Researcher in Health Economics
Senior Researcher in Health Economics
Health Economics & Design Science
(ScHARR)
University of Sheffield
Sheffield.
UK

Mr Mostafa Metwally MD FRCOG - Consultant Gynaecologist and Subspecialist in Reproductive Medicine and Surgery
Fertility Unit - Jessop Wing
Sheffield Teaching Hospitals NHS Foundation Trust
Sheffield.
UK

For peer review only

For peer review only - http://bmjopen.bmj.com/site/about/guidelines.xhtml

Key words:

Endometrial Scratch

In Vitro Fertilisation

Endometrial Trauma

Assisted Conception

Randomised Controlled Trial

Abstract

Introduction

Endometrial Trauma commonly known as Endometrial Scratch (ES) has been shown to improve pregnancy rates in women with a history of repeated implantation failure undergoing In Vitro Fertilisation (IVF), with or without Intracytoplasmic Sperm Injection (ICSI). However, the procedure has not yet been fully explored in women having IVF/ICSI for the first time. This study aims to examine the effect of performing an ES in the mid-luteal phase prior to first time IVF/ICSI cycle on the chances of achieving a clinical pregnancy and live birth. If ES can influence this success rate there would be a significant cost saving to the NHS through decreasing the number of IVF/ICSI cycles necessary to achieve a pregnancy, increase the practice of single embryo transfer (SET) and consequently have a large impact on risks and costs associated with multiple pregnancies.

Methods & Analysis

This 30 month, UK, multi-centre, parallel group, RCT includes a 9month internal pilot and health economic analysis recruiting 1044 women from 16 Fertility Units. It will follow up participants to identify if IVF/ICSI has been successful and live birth has occurred up to 6 weeks post-partum. Primary analysis will be on an intention to treat basis. A sub-study of endometrial samples obtained during the ES will assess the role of immune factors in embryo implantation. Main trial recruitment commenced January 2017 and is ongoing.

Participants randomised to the intervention group will receive the ES procedure in the mid luteal phase of the preceding cycle prior to first time IVF/ICSI treatment versus usual IVF/ICSI treatment in the control group, with 1:1 randomisation. The primary outcome is live birth rate (LBR) after completed 24 weeks gestation.

Ethics and dissemination

South Central – Berkshire NREC approved the protocol. Findings will be submitted to peer-reviewed journals and abstracts to relevant national and international conferences.

Trial Registration number: ISRCTN: 23800982. Protocol Date: Version 5 Dated 20/07/2017

For peer review only - http://bmjopen.bmj.com/site/about/guidelines.xhtml

Strengths and limitations of this study

- This is the largest multicentre, pragmatic randomised controlled trial to date which aims to assess the effectiveness and cost effectiveness of performing the ES procedure in women having IVF/ICSI for the first time
- It aims to determine whether performing an ES is an acceptable and well tolerated procedure.
- Due to the nature of the intervention it is not possible to blind study participants or clinicians.
- Potential difficulty with recruitment if patients are not in equipoise about effectiveness of the ES procedure in first time IVF/ISCI cycles.

Background

The use of local endometrial trauma known as Endometrial Scratch (ES) to improve implantation rates in women undergoing assisted conception was first described in 2003 [1]. The procedure has since been explored in several studies mainly focusing on women with recurrent implantation failure and has been shown to significantly increase pregnancy rates by almost double [2–4]. However, uncertainty remains as to the therapeutic effect of ES, due to heterogeneity of the populations included - and the timing and exact protocol of ES used - in previous evaluations [5,6]. Three systematic reviews have summarised the evidence, however each included different studies [2,7,8]. A recent Cochrane review included fourteen randomised studies; seven in women with previous cycle failure, five in an unselected population and one in a first-time cycle [8]. The live birth rate meta-analysis combined trials regardless of the population (i.e. number of previous IVF cycles) and included five studies, reporting a risk ratio (RR) of 1.42 (1.08, 1.85), p=0.02. The odds of achieving a clinical pregnancy were also increased following ES with a RR of 1.34 (1.11, 1.62), p=0.002. The one trial conducted in women undergoing their first IVF cycle indicated the procedure was harmful with an OR of clinical pregnancy rate of 0.30 (0.14, 0.63) p=0.002 [9]. Notably, this trial performed the ES procedure at the time of oocyte retrieval and not in the month prior to the IVF cycle. Despite the concerns around the quality of evidence in using ES and that many of the trials undertaken so far have been small (most <150 participants), ES has been widely adopted into routine clinical practice in women with recurrent unsuccessful implantation and is currently being provided in some fertility units where women are having IVF/ICSI for the first time [10,11]. Two large trials are currently in progress to determine if ES is beneficial in women undergoing their 2nd IVF cycle [12] and a sample of women undergoing any IVF cycle [13]. Therefore, given the lack of evidence for the effectiveness of ES in women undergoing their 1st cycle of IVF/ICSI, it is essential that a large well controlled multi-centre trial is conducted to fully investigate the effectiveness and safety of this technique.

The Human Fertilisation and Embryology Authority (HFEA) state in their statistical report into multiple births that the risks associated with multiple births is the single biggest health risk associated with fertility treatment [14]. Multiple births carry risks to the health of both

For peer review only - http://bmjopen.bmj.com/site/about/guidelines.xhtml

the mother and the babies and that birth of a healthy singleton child, born at full term, is therefore the safest outcome of fertility treatment for both mother and child and is best achieved through promoting the practice of SET.

If ES can improve the implantation potential of the embryo and therefore improve success rates, ES may encourage an expansion of current SET policies. Inclusion of women with a lower chance of having cryopreserved embryos and a more general increase in the implementation of the practice of SET, could consequently have a large impact on the risks and costs associated with multiple pregnancies as a result of IVF [15].

The exact mechanism by which ES may improve implantation is not yet known, however it is known that implantation is a complex process involving a number of inflammatory mediators including uterine natural killer cells, leukaemia inhibitory factor and interleukin 15 [13]. It is possible that ES may lead to the activation of inflammatory cells such as macrophages and dendritic cells, and release of inflammatory mediators such as tumour necrosis factor-α, interleukin-15, growth-regulated oncogene-α and macrophage inflammatory protein 1B [14].

ES has also been shown to cause the modulation of several endometrial genes that may be involved in membrane stability during the process of implantation such as bladder transmembranal protein (UPIb) and adipose differentiation-related protein and mucin 1 [16].

ES is routinely performed as an outpatient procedure. Risks have been identified in a previous study when the procedure was undertaken on the day of oocyte retrieval (reduced implantation and pregnancy rates) [9]; however, the procedure is not known to be associated with any particular risks when undertaken in the menstrual cycle preceding that of IVF therapy, apart from period like discomfort whilst performing the procedure. Taking simple analgesics prior to the procedure usually alleviates this. As with any intrauterine procedure there is a potential for intrauterine infection. However women attending for fertility treatment are usually screened for serious vaginal infections such as chlamydia to minimise the risk of any spread of infection when performing the embryo transfer procedure, a similar procedure to an ES as it involves the insertion of a catheter into the uterine cavity.

The main objectives of this trial are to assess the clinical and cost effectiveness of the ES procedure in women aged between 18 and 37 years (inclusive) undergoing their first IVF/ICSI cycle using either antagonist or long protocols to see if it could potentially improve implantation rates and hence encourage the practice of single embryo replacement. A sub-study will be undertaken in two of the fertility units where endometrial samples obtained from the ES procedure will be stored for later analysis to identify endometrial factors associated with successful pregnancy outcome.

For peer review only - http://bmjopen.bmj.com/site/about/guidelines.xhtml

Method and Analysis

The Endometrial Scratch Trial is a multi-centre, parallel group, randomised controlled trial to examine the clinical, cost effectiveness and safety of an ES performed in the mid-luteal phase of the preceding cycle prior to a first time IVF/ICSI cycle. Eligible participants will be randomised to either the treatment as usual (TAU) arm, consisting of usual IVF treatment, or the intervention arm where ES will be performed followed by usual IVF treatment. The overall study design is illustrated below in the study flow chart (figure 1).

Patient and Public Involvement

The study was reviewed by couples waiting to commence IVF treatment and then by the members of The Jessop Wing Reproductive Health Public Advisory Panel (PPI) at the Jessop Wing-Sheffield. All were asked to provide input into the lay summary, recruitment strategy, visit schedule and benefits of the proposed study to the patient & the NHS. We asked about their experience of assisted conception, the things they liked and disliked, and the potential difficulties or barriers to attending for treatment, randomisation to the TAU arm and how this might affect recruitment but clarified that if the trial showed an increase in the scratch arm assisting embryo implantation, then it would form part of the routine care pathway in the future. A member of the panel agreed to become the Service User Representative, is a member of the Steering committee and attended the Trial Investigator/set-up meeting providing a patients view of all aspects associated with IVF. All PPI members have provided input into the patient facing documents on an ongoing basis and prior to submission to ethics and are aware of recruitment and the conduct of the study at ongoing PPI events held on a bimonthly basis within the Directorate.

The most significant changes to the HTA grant influenced by the PPI members were in relation to trial follow-up procedures as they felt only women who achieved a pregnancy should be followed up. They also wanted to ensure continuity across the participating centres when performing the follow-up visits and requested a proforma be designed to ensure all research nurses/midwives capture the same information.

Upon completion of the trial the results will be summarised in plain English and distributed to participants and patient support groups such as Infertility Network UK with the assistance of our service-user collaborators. We will promote the transfer of knowledge to wider audiences including the general public (e.g. including short, user-friendly articles/briefings in relevant newsletters, magazines and periodicals, user groups/forums).

Figure 1. Study flow chart.

The trial consists of two phases - an internal pilot to assess feasibility of recruitment and delivery of the intervention, and a two year main recruitment phase.

For peer review only - http://bmjopen.bmj.com/site/about/guidelines.xhtml

The trial will commence with a 9 month internal pilot recruitment phase across approximately 6 UK fertility units to justify whether or not the recruitment strategy and the scheduling of the endometrial scratch procedure are feasible and will use the same trial procedures as described for the main trial.

The trial is collaboration between research staff at The Jessop Wing, Sheffield Teaching Hospital NHS Foundation Trust & The University of Sheffield - Clinical Trials Research Unit (CTRU) who is responsible for the conduct of the trial. Funding to run the trial has been awarded by the National Institute of Healthy Research (NIHR) Health Technology Assessment (HTA). At the end of the pilot phase, the Trial Steering Committee (TSC) will report to the funder on whether the feasibility criteria have been met and whether the trial should continue.
The trial will be conducted in compliance with the approved protocol, GCP and regulatory requirements. Main trial recruitment commenced January 2017 and is ongoing.

Sheffield CTRU will aggregate feasibility of the research and intervention protocols based on the following outcomes.
The trial will be considered infeasible and will be stopped if either of the following conditions apply:

1. Feasibility of recruitment to the main trial: defined as recruitment of fewer than 108 participants (75% of the 144 target) during the internal pilot phase.

2. Scheduling of the ES procedure: defined as less than 75% of women scheduled to receive their ES procedure have received the ES at the correct time point.

Recruitment
Upon successful completion of the pilot the main trial will aim to recruit women from 16 UK Fertility Units requiring first time IVF treatment. Participation is entirely voluntary and choosing not to participate will not negatively influence the woman's treatment in any way. Furthermore consent can be withdrawn at any stage. Women who are about to undergo their first cycle of IVF/ICSI will be identified by screening patients referred for these treatments. Eligible women will be sent information regarding the trial in the post or via e-mail or may be alerted to the trial via the trial website or posters displayed at the fertility unit. If they are interested in participating they will be invited to discuss the trial with their fertility team at their next routine appointment.

Prior to randomisation full written informed consent will be obtained by a suitably trained Doctor or Research Nurse/Midwife at a clinic visit. The participant will complete a study specific resource use questionnaire prior to randomisation to collect health care usage in the previous 3 months; baseline data will be collected at this visit and participants will be randomly allocated to either the intervention or usual care arm of the trial.

For peer review only - http://bmjopen.bmj.com/site/about/guidelines.xhtml

Detailed methods of the Endometrial Scratch trial are described in the Endometrial Scratch protocol available on the website – https://www.sheffield.ac.uk/scratchtrial

Women will be included and considered suitable if they meet the following eligibility criteria:

Inclusion criteria

1. Women expected to be aged between 18 and 37 years (inclusive) at time of egg collection.
2. First time IVF with or without ICSI treatment using the antagonist or long protocol only.
3. Expected to receive treatment using fresh embryos.
4. Expected good responders to treatment, with:

a. Ovulatory menstrual cycle (Regular menstrual cycles defined by clinical judgement or with ovulatory levels of mid-luteal serum progesterone as defined by local laboratory protocols)

b. Normal uterine cavity (assessed by transvaginal sonography at screening and no endometrial abnormalities such as , suspected intrauterine adhesions, uterine septa, submucosal fibroids or intramural fibroids exceeding 4 cm in diameter as assessed by the investigator that would require treatment to facilitate pregnancy).

c. Expected good ovarian reserve (assessed clinically, biochemically (FSH< 10 & normal follicular phase oestradiol levels and or normal AMH), and or sonographically (antral follicle counts) and no history of previous radiotherapy or chemotherapy). [All laboratory/ultrasound standards based on local normal reference ranges.]

d. Single embryo transfer (SET) expected.

5. Local procedures have been / will be followed to exclude relevant vaginal/uterine infections prior to starting treatment.
6. Willing to use an appropriate method of barrier contraception if randomised to Endometrial Scratch (ES) in the cycle where the ES procedure is performed. .
7. Understands/willing to comply with the protocol.

Exlcusion criteria

1. Previous trauma/surgery to the endometrium (e.g. resection of submucous fibroid, intrauterine adhesions.).
2. BMI of 35 kg/m2 or greater.
3. Known grade 4 (severe) endometriosis.
4. Currently participating in any other fertility study involving medical/surgical intervention.
5. Expected to receive protocols other than antagonist or long (e.g. ultra-long protocol).
6. An endometrial scratch (or similar procedure, e.g. endometrial biopsy for the collection of Natural Killer Cells) is planned.
7. Previously randomised into this trial.

For peer review only - http://bmjopen.bmj.com/site/about/guidelines.xhtml

Sampling

The primary outcome is the LBR. This is defined as a live birth after completed 24 weeks gestation, within the 10.5 month post egg collection follow-up period.This time-period will enable the collection of any neonatal deaths (up to 6 weeks post-partum). The denominator for calculating the LBR will be the number of women randomised to each group. Data from the HFEA suggests a live birth rate of 32.8% in women under 35 and 27.3 % in women aged 35-37. The sample size calculation assumes a 30% LBR in the control group and that an absolute increase of 10%, to a 40% LBR (a relative risk of 1.33) in the intervention groups is of clinical and practical importance. The effect size, a 10% absolute difference in LBR, we are proposing is large but we believe an effect of such magnitude is needed to change clinical practice (there is a 5% absolute difference in LBR between women aged under 35 and 35-37) and is less than that observed in the systematic reviews described above (where, at the time of sample size calculation, the relative risk estimates for live birth ranged from 1.83 to 2.46) [2,17]. To have a 90% power of detecting this difference or more, in LBR rates between the groups, as statistically significant at the 5% two-sided level, will require 496 women per group (992 in total). Adjusting for a predicted drop-out rate of 5% (due to anticipated difficulties of follow-up for patients who have been referred from NHS Trusts other than the participating Fertility Unit) we will require 1044 participants.

Study procedures

Following randomisation women in the intervention arm will have the ES procedure performed in the mid-luteal phase of the preceding cycle prior to their planned IVF/ICSI cycle in the outpatient setting of the fertility unit. The choice of screening for infection prior to the procedure or the administration of antibiotics will be left to individual unit according to their local established protocols and procedures. Women can be randomised any time up until they start their IVF cycle, although it may be necessary for the participant to delay her IVF if randomised to the intervention arm. This decision to delay should be made and agreed by both the patient and her fertility team before randomisation is undertaken. The oral contraceptive pill can be used following randomisation for the purposes of cycle programming but women must be having ovulatory periods at the point of entry into the trial. Women randomised to TAU will continue with their IVF/ICSI as planned and will not receive the ES procedure.

Following delivery of the ES, participants will undergo IVF/ICSI in line with local procedures. Following successful embryo transfer (in both groups) a pregnancy test will be performed and adverse events will be collected. In cases where women do not undergo embryo transfer, every effort will be made by the research team to collect any adverse event information from either the patient or the medical notes. If a pregnancy is confirmed the woman is discharged to normal antenatal care as per standard practice. It is the intention to obtain the pregnancy status of all women once randomised including the outcome of all spontaneous pregnancies, the first frozen embryo transfer if no fresh transfer has been undertaken as well as those that delay treatment following the ES. Women will not be

For peer review only - http://bmjopen.bmj.com/site/about/guidelines.xhtml

followed-up if they withdraw their consent from the trial. Data will also be collected regarding participants who have received an ES outside the trial.

Randomisation

The randomisation schedule will be generated by Sheffield CTRU prior to the start of the trial; access to the schedule will be limited only to the trial statistician. The randomisation sequence will be computer generated and stratified by site and protocol (antagonist or long protocol). Random permuted blocks of variable size will be used to ensure enough participants are allocated evenly to each arm of the trial at each site. Research staff at recruiting centres will be unable to access the randomisation sequence and will use a web based computer system with restricted access rights to enter participant details; randomisation outcome will then be revealed. Re-randomisation will not be permitted.

Trial Intervention

ES is a minor procedure of 10 to 20 minute duration that will be performed in an outpatient setting at local IVF centres in line with local procedures and the trial standard operation procedure (SOP). The participant will be required to use a barrier method of contraception (if necessary) during the menstrual cycle in which the ES will be performed. During ES, a speculum is inserted into the vagina and the cervix exposed and cleaned. A pipelle or similar endometrial sampler is then inserted into the cavity of the uterus; negative pressure is applied by withdrawal of the plunger. The sampler is rotated and withdrawn several times so that tissue appears in the transparent tube. The sampler and speculum are then removed. If no tissue is seen in the transparent sampler, this is an indication that the sampler was not fully inside the uterine cavity and therefore the procedure is repeated. Following the procedure women will complete a visual pain scale (likert) to assess their pain and tolerability assessment of the procedure within 30 minutes of the initial ES and then again at 24 hours and 7 days post procedure via an automated text message.

Compliance to the intervention will be ascertained through the clinician or Research Nurse/Midwife recording whether or not the patient has a) attended the clinic for the ES procedure and b) received the ES procedure as per protocol. Any deviation from the protocol will be noted and reported as per the Sheffield CTRU SOP.

Follow-up

Patient follow up will continue until either the 1st cycle of IVF has been completed or the resulting pregnancy has concluded. If no pregnancy is confirmed the study is complete (regardless of which group the woman is randomised to). Pregnant women will be followed-up at 3 and 6 months post egg collection and then 6 weeks post-partum to collect pregnancy outcome and safety data. If the pregnancy is ongoing at 3 months and 6 week post-partum, a health resource use questionnaire will be sent to the patient for completion. If a spontaneous pregnancy is achieved between randomisation and IVF treatment, the pregnancy will be followed up as described above, instead of egg collection, the date of the

For peer review only - http://bmjopen.bmj.com/site/about/guidelines.xhtml

last menstrual period (LMP) will be used to schedule the 3/6 month & 6 week post-partum follow ups.

Safety considerations, safety monitoring and AE reporting

All Adverse Events (AEs) and Serious Adverse Events (SAEs) will be recorded by the local research team at each Fertility Unit. All AEs/SAEs will be followed up until satisfactory resolution or until the treating Clinician and the Principal Investigator deems the event to be chronic or the participant to be stable. Research Nurses/Midwives will ask patients for any details of adverse events at five time-points: post procedure (if randomised to receive ES), at the participants' pregnancy test, and then, if pregnancy has been achieved, at 3 and 6 months post egg collection and finally 6 weeks post-partum.

AEs/SAEs will be collected up to the participants' final study related follow-up event. If embryo transfer does not occur, the Research Nurse/Midwife will contact the participant approximately 2 weeks after egg collection to identify if any adverse events have occurred. In the case of a negative pregnancy test, the site research team should make every effort to obtain AE data from the patient or the medical notes at routine clinical care contacts; no further contact will be made outside of routine clinical care.

Expected AEs will be those which occur regularly due to pregnancy, and expected SAEs are those events which are expected in the patient population as a result of the routine care/treatment of a patient. Expected SAEs and all AEs will be collected as part of the trial and entered into the eCRF, but will not be reported to regulatory bodies (NHS REC/sponsor). Unexpected SAEs will be reported to the Sheffield CTRU as soon as staff at the fertility units become aware of the event.

All SAEs will be reviewed by the Data Monitoring & Ethics Committee (DMEC) and Trial Management Group (TMG) at regular intervals. The Chief Investigator (CI) will inform all Principal Investigators (PIs) concerned of relevant information that would adversely affect the safety of the participants.

Outcomes

Primary clinical outcome

- Live birth rate; based on the number of live births after 24 weeks gestation within the 10.5 month post egg collection follow-up period.

Secondary outcomes

- Acceptability and pain rating of the Endometrial Scratch procedure, a visual pain scale (likert) to assess their pain and tolerability assessment of the procedure within 30minutes of the initial ES procedure, 1 day later and then again 7 days after the ES.
- Implantation rate based on a positive serum Beta hCG on approximately day 14 following the egg collection or by a positive urine pregnancy test.

For peer review only - http://bmjopen.bmj.com/site/about/guidelines.xhtml

- Clinical pregnancy rate; an observation of viable intrauterine pregnancy with a positive heart pulsation seen on ultrasound at/after 8 weeks gestation
- Miscarriage rate as measured by spontaneous pregnancy loss (including pregnancy of unknown location (PUL) prior to 24 weeks gestation within the 10.5 month post egg collection follow-up period
- Ectopic pregnancy as measured by the rate of pregnancy outside the normal uterine cavity
- Multiple birth rate, defined as the birth of more than one living foetus after completed 24 weeks gestation
- Preterm delivery rate as measured by live birth after 24 weeks before 37 weeks gestation within the 10.5 month post egg collection follow-up period.
- Still birth rate based on the delivery of a still born foetus showing no signs of life after 24 weeks gestation within the 10.5 month post egg collection follow-up period.
- Details of participant's IVF cycles including number of eggs retrieved, number of embryos generated 1 day after egg collection, quality of the embryos transferred (using NEQAS grading) and the number of embryos replaced and day of embryo replacement.
- Adverse events
- Health resource use of the participant & patient costs

The trial includes a health economic component to assess the cost of the intervention per extra live birth from an NHS and social care perspective. Resource use will include the intervention costs for ES, the cost of IVF treatment, visits to the Assisted Conception Unit and for those who conceive antenatal and post-natal visits, delivery costs and any hospital stays not related to birth for both mother and baby. The resource use questionnaire will collect information on contacts with midwife and GP visits. A Patient Cost questionnaire will collect time taken to travel to appointments and loss of productivity. Unit costs will be derived from appropriate national sources and will include; NHS reference costs, Personal Social Service Research Unit costs and the **Office of National Statistics** [18–20]. The resource use questionnaire will be designed for this study and will draw on data collection tools developed in The School of Health and Related Research (ScHARR) and those collated by the Database for Instruments for Resource Use Measurement (DIRUM).

Blinding

Since this trial evaluates objectively measured outcomes (pregnancy rates) that are unlikely to be affected by a placebo effect, participants will not be blinded to treatment allocation; it is therefore not necessary to perform a sham procedure for the control group. The study statistician, TSC and health economist will be blinded to the allocation.

Trial monitoring and oversight committees

The trial will be overseen by the TSC and the DMEC, membership of both will consist of independent experts in the field. The TSC will include a patient representative. Both

For peer review only - http://bmjopen.bmj.com/site/about/guidelines.xhtml

committees will review recruitment, study progress and adverse events. The DMEC will receive monthly reports of recruitment and adverse events and, at their meetings, will also consider emerging evidence from other trials or research on ES. They may advise the chair of the TSC at any time if, in their view, the trial should be stopped for ethical reasons, including concerns about patient safety.

Day-to-day running of the trial will be coordinated by the Trial Management Group (TMG), consisting of the grant co-applicants, plus members of the Jessop Wing Fertility Unit, Sheffield CTRU and Patient representatives.

Statistical analysis

Primary analysis will be performed on the intention to treat population (all participants randomised into the trial). All statistical exploratory tests will be two-tailed at 5% nominal level. Baseline demographic (e.g. age), physical measurements (e.g. BMI), and health-related data will be described and summarised overall and for both treatment groups. The women, not the IVF cycle will be the unit of analysis. If the woman fails to get pregnant or does not have IVF treatment, they will be included in the analysis of the primary outcome as a negative outcome (i.e. non-live birth). For sensitivity analyses, per protocol (PP) analyses will also be undertaken which will be defined as for Endometrial Scratch participants in the intervention group, receiving the ES procedure as documented in the study protocol and undergoing IVF/ICSI in the subsequent menstrual cycle, including embryo transfer. For the control group, the PP population will receive IVF/ICSI including embryo transfer. Sub-group analyses will be undertaken to explore the effect of important variables related to the participant and their treatment on the primary and secondary outcomes. These subgroups are:

- Day of embryo transfer (day 2, 3, 4, 5 or 6),
- Fertilisation method (IVF, IVF or ICSI, ICSI [spilt]),
- Type of protocol (long or antagonistic),
- Embryo transfer (single or double) and whether the embryo was fresh or frozen
- Previous history of consecutive miscarriages (0-2 vs >=3)

AEs will be reported as a proportion of all women randomised. Adverse events including serious adverse events will be compared between the two groups using a Fisher's Exact test, Chi-squared test or negative binomial regression model in case of repeated events per woman (as appropriate). A 95% CI for the difference in adverse event rate between the groups will also be calculated with associated point estimate depending on the method used.

Health economic results will be presented in the net-benefit framework and will allow for uncertainty using bootstrapping and probabilistic sensitivity analysis.

For peer review only - http://bmjopen.bmj.com/site/about/guidelines.xhtml

Ethics and dissemination

The study is registered on the ISRCTN database (reference 23800982) and has been approved by the South Berkshire Research Ethics Committee (reference 16/SC/0151). The findings of this trial will be submitted to peer- reviewed journals and abstracts to national and international conferences. Other stakeholder specific outputs in relevant formats will also be produced for commissioners, IVF practitioners, third sector and user advocacy organisations. A website will be established to promote the work of the trial. All knowledge transfer activity including translation will be informed by input from trial collaborators, the TSC and TMG to ensure the study is meeting the needs of the commissioners and audience.

Discussion

This trial will determine whether performing an ES procedure prior to 1st time IVF/ICSI treatment is an inexpensive, safe and well tolerated procedure that increases the live birth rate in women having SET. If shown to be the case, this will have a significant improvement in first cycle IVF success rates and potentially lead to significant cost savings to the NHS as fewer women would need to have repeat treatment cycles. This is particularly important in the current economic climate and with restrictions on funding and service provision. This will also have a significant impact for women, for whom the burden of repeated cycles is large.

Acknowledgements

We thank Dr Munya Dimairo - Research Fellow in Medical Statistics at the University of Sheffield for his contribution to the design/content of the protocol, The Jessop Wing Reproductive Health Public Advisory Panel as well as each local PI, Research Nurse/Midwife and research delivery team in the recruitment and management of patients.

Authors Contributions

Contributors: MM, CP, JC, KB, YC, SL, LM, JS, SW &, TY conceived the study, and contributed to study design, sample size calculations and analytical plans.

MM, CP, RC, JC, KB, YC, SL, LM, JS, SW & TY initiated the project, have assisted in developing the protocol and helped with implementation.

CP, RC, JC, & MM drafted the manuscript. All authors read and approved the final manuscript.

Competing interests

None declared.

Funding statement

This trial was funded by the NIHR Health Technology Assessment program grant number [14/08/45]

For peer review only - http://bmjopen.bmj.com/site/about/guidelines.xhtml

This article presents independent research supported by the National Institute for Health Research (NIHR). The views expressed are those of the author(s) and not necessarily those of the NHS, the NIHR or the Department of Health

References

1 Barash A, Dekel N, Fieldust S, *et al.* Local injury to the endometrium doubles the incidence of successful pregnancies in patients undergoing in vitro fertilization. *Fertil Steril* 2003;**79**:1317–22. doi:10.1016/S0015-0282(03)00345-5

2 El-Toukhy T, Sunkara S, Khalaf Y. Local endometrial injury and IVF outcome: A systematic review and meta-analysis. *Reprod Biomed Online* 2012;**25**:345–54. doi:10.1016/j.rbmo.2012.06.012

3 Raziel A, Schachter M, Strassburger D, *et al.* Favorable influence of local injury to the endometrium in intracytoplasmic sperm injection patients with high-order implantation failure. *Fertil Steril* 2007;**87**:198–201. doi:10.1016/j.fertnstert.2006.05.062

4 Huang SY, Wang C-J, Soong Y-K, *et al.* Site-specific endometrial injury improves implantation and pregnancy in patients with repeated implantation failures. *Reprod Biol Endocrinol* 2011;**9**:140. doi:10.1186/1477-7827-9-140

5 Simón C, Bellver J. Scratching beneath 'the Scratching Case': Systematic reviews and metaanalyses, the back door for evidencebased medicine. Hum. Reprod. 2014;**29**:1618–21. doi:10.1093/humrep/deu126

6 Santamaria X, Katzorke N, Simón C. Endometrial 'scratching': What the data show. Curr. Opin. Obstet. Gynecol. 2016;**28**:242–9. doi:10.1097/GCO.0000000000000279

7 Potdar N, Gelbaya T, Nardo LG. Endometrial injury to overcome recurrent embryo implantation failure: a systematic review and meta-analysis. *Reprod Biomed Online* 2012;**25**:561–71. doi:10.1016/j.rbmo.2012.08.005

8 Nastri CO, Lensen SF, Gibreel A, *et al.* Endometrial injury in women undergoing assisted reproductive techniques. *Cochrane database Syst Rev* 2015;**3**:CD009517. doi:10.1002/14651858.CD009517.pub3

9 Karimzade MA, Oskouian H, Ahmadi S, *et al.* Local injury to the endometrium on the day of oocyte retrieval has a negative impact on implantation in assisted reproductive cycles: A randomized controlled trial. *Arch Gynecol Obstet* 2010;**281**:499–503. doi:10.1007/s00404-009-1166-1

10 Spencer EA, Mahtani KR, Goldacre B, *et al.* Claims for fertility interventions: a systematic assessment of statements on UK fertility centre websites. *BMJ Open* 2016;**6**:e013940. doi:10.1136/bmjopen-2016-013940

11 Lensen S, Sadler L, Farquhar C. Endometrial scratching for subfertility: Everyone's doing it. *Hum Reprod* 2016;**31**:1241–4. doi:10.1093/humrep/dew053

For peer review only - http://bmjopen.bmj.com/site/about/guidelines.xhtml

12 van Hoogenhuijze NE, Torrance HL, Mol F, *et al.* Endometrial scratching in women with implantation failure after a first IVF/ICSI cycle; does it lead to a higher live birth rate? The SCRaTCH study: A randomized controlled trial (NTR 5342). *BMC Womens Health* 2017;**17**. doi:10.1186/s12905-017-0378-y

13 Lensen S, Martins W, Nastri C, *et al.* Pipelle for Pregnancy (PIP): study protocols for three randomised controlled trials. *Trials* 2016;**17**:216. doi:10.1186/s13063-016-1301-9

14 Human Fertilisation & Embryology Authority. Improving outcomes for fertility patients: Multiple births. A statistical report. 2011.

15 Ledger WL, Anumba D, Marlow N, *et al.* The costs to the NHS of multiple births after IVF treatment in the UK. *BJOG* 2006;**113**:21–5. doi:10.1111/j.1471-0528.2005.00790.x

16 Kalma Y, Granot I, Gnainsky Y, *et al.* Endometrial biopsy-induced gene modulation: first evidence for the expression of bladder-transmembranal uroplakin Ib in human endometrium. *Fertil Steril* 2009;**91**:1042–9, 1049-9. doi:10.1016/j.fertnstert.2008.01.043

17 Nastri CO, Gibreel A, Raine-Fenning N, *et al.* Endometrial injury in women undergoing assisted reproductive techniques. *Cochrane database Syst Rev* 2012;**7**:CD009517. doi:10.1002/14651858.CD009517.pub2

18 NHS reference costs 2012/13. 2013.

19 Curtis L. Unit costs of health and social care 2013. 2013.

20 Annual survey for hours and earnings (ASHE). 2012. http://www.ons.gov.uk/ons/rel/ashe/annual-survey-of-hours-and-earnings/2012-revised-results/index.html (accessed 12 Sep 2014).

For peer review only

For peer review only - http://bmjopen.bmj.com/site/about/guidelines.xhtml

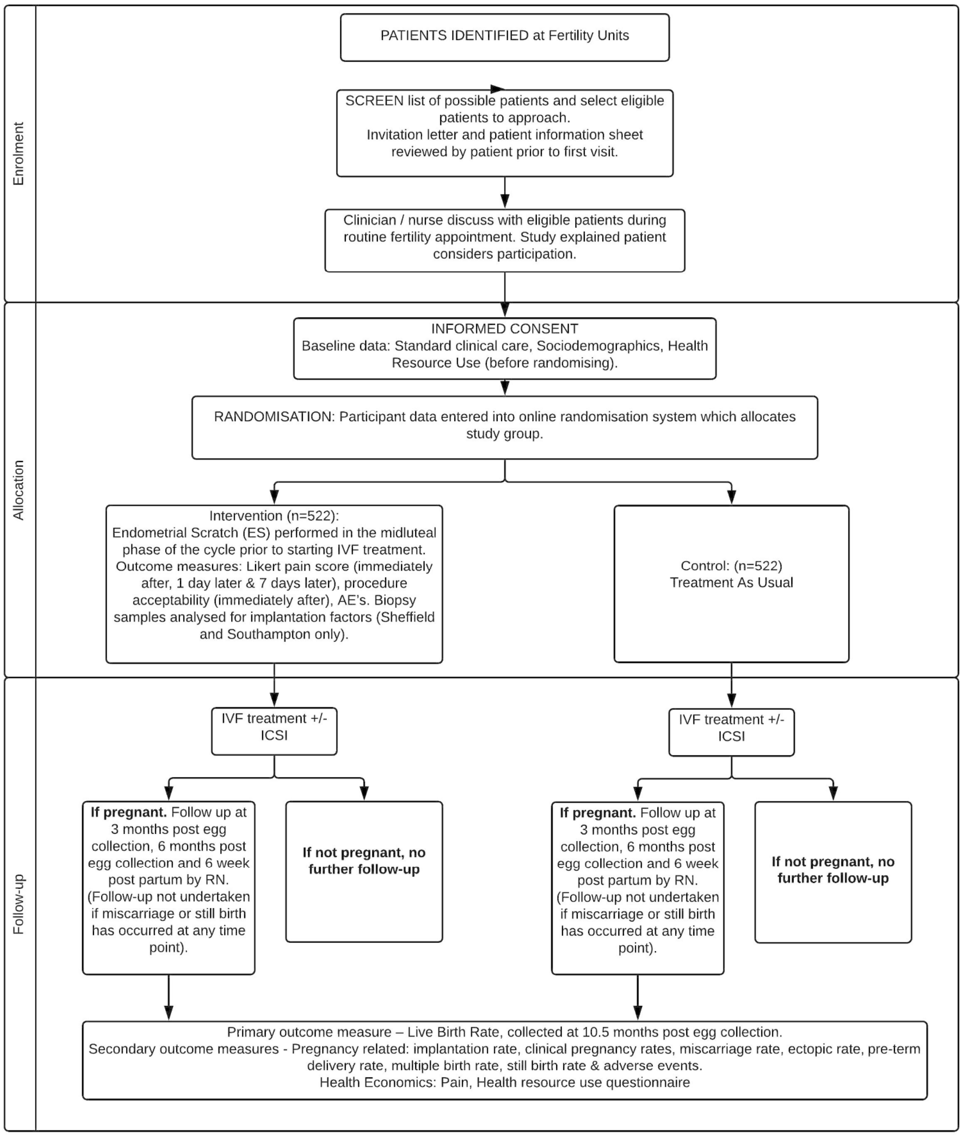

203x241mm (300 x 300 DPI)

SPIRIT
STANDARD PROTOCOL ITEMS: RECOMMENDATIONS FOR INTERVENTIONAL TRIALS

SPIRIT 2013 Checklist: Recommended items to address in a clinical trial protocol and related documents*

Section/item	Item No	Description	Addressed on page number
**Administrative information**			
Title	1	Descriptive title identifying the study design, population, interventions, and, if applicable, trial acronym	Pages 1, 3 & 4 in Manuscript<br>Protocol Version 5 Dated 20/07/2017
Trial registration	2a	Trial identifier and registry name. If not yet registered, name of intended registry	Page 3 in manuscript<br>Protocol Version 5 Dated 20/07/2017
	2b	All items from the World Health Organization Trial Registration Data Set	Page 3/4 in Manuscript<br>Protocol Version 5 Dated 20/07/2017
Protocol version	3	Date and version identifier	Page 3 in Manuscript.<br>Protocol Version 5 Dated 20/07/2017

For peer review only - http://bmjopen.bmj.com/site/about/guidelines.xhtml

Funding	4	Sources and types of financial, material, and other support	Page 7 & 14 in Manuscript Protocol Version 5 Dated 20/07/2017
Roles and responsibilities	5a	Names, affiliations, and roles of protocol contributors	Page 1, 2 & 14 in Manuscript Protocol Version 5 Dated 20/07/2017
	5b	Name and contact information for the trial sponsor	Protocol Version 5 Dated 20/07/2017
	5c	Role of study sponsor and funders, if any, in study design; collection, management, analysis, and interpretation of data; writing of the report; and the decision to submit the report for publication, including whether they will have ultimate authority over any of these activities	Page 14
	5d	Composition, roles, and responsibilities of the coordinating centre, steering committee, endpoint adjudication committee, data management team, and other individuals or groups overseeing the trial, if applicable (see Item 21a for data monitoring committee)	Pages 7, 12 & 13 in Manuscript. Protocol Version 5 Dated 20/07/2017
**Introduction**			
Background and rationale	6a	Description of research question and justification for undertaking the trial, including summary of relevant studies (published and unpublished) examining benefits and harms for each intervention	Pages 3, 4 & 5 in Manuscript Protocol Version 5 Dated 20/07/2017
	6b	Explanation for choice of comparators	Page 6 in Manuscript Protocol Version 5 Dated 20/07/2017

For peer review only

Objectives	7	Specific objectives or hypotheses	Page 3 in Manuscript Protocol Version 5 Dated 20/07/2017
Trial design	8	Description of trial design including type of trial (eg, parallel group, crossover, factorial, single group), allocation ratio, and framework (eg, superiority, equivalence, noninferiority, exploratory)	Pages 3, 4, 5 & 6 in Manuscript Protocol Version 5 Dated 20/07/2017
**Methods: Participants, interventions, and outcomes**			
Study setting	9	Description of study settings (eg, community clinic, academic hospital) and list of countries where data will be collected. Reference to where list of study sites can be obtained	Pages 3 & 7 in Manuscript Protocol Version 5 Dated 20/07/2017
Eligibility criteria	10	Inclusion and exclusion criteria for participants. If applicable, eligibility criteria for study centres and individuals who will perform the interventions (eg, surgeons, psychotherapists)	Page 8 in Manuscript Protocol Version 5 Dated 20/07/2017
Interventions	11a	Interventions for each group with sufficient detail to allow replication, including how and when they will be administered	Page 9 & 10 in Manuscript Protocol Version 5 Dated 20/07/2017
	11b	Criteria for discontinuing or modifying allocated interventions for a given trial participant (eg, drug dose change in response to harms, participant request, or improving/worsening disease)	Page 10 in Manuscript Protocol Version 5 Dated 20/07/2017
	11c	Strategies to improve adherence to intervention protocols, and any procedures for monitoring adherence (eg, drug tablet return, laboratory tests)	Page 10 & 11 in Manuscript Protocol Version 5 Dated 20/07/2017

For peer review only - http://bmjopen.bmj.com/site/about/guidelines.xhtml

	11d	Relevant concomitant care and interventions that are permitted or prohibited during the trial	Page 10 & 11 in Manuscript Protocol Version 5 Dated 20/07/2017
Outcomes	12	Primary, secondary, and other outcomes, including the specific measurement variable (eg, systolic blood pressure), analysis metric (eg, change from baseline, final value, time to event), method of aggregation (eg, median, proportion), and time point for each outcome. Explanation of the clinical relevance of chosen efficacy and harm outcomes is strongly recommended	Page 11 & 12 in Manuscript Protocol Version 5 Dated 20/07/2017
Participant timeline	13	Time schedule of enrolment, interventions (including any run-ins and washouts), assessments, and visits for participants. A schematic diagram is highly recommended (see Figure)	Pages 6, 7 & 10 in Manuscript Protocol Version 5 Dated 20/07/2017
Sample size	14	Estimated number of participants needed to achieve study objectives and how it was determined, including clinical and statistical assumptions supporting any sample size calculations	Page 3, 7 & 9 in Manuscript Protocol Version 5 Dated 20/07/2017
Recruitment	15	Strategies for achieving adequate participant enrolment to reach target sample size	Page 7 & 8 in Manuscript Protocol Version 5 Dated 20/07/2017

Methods: Assignment of interventions (for controlled trials)

Allocation:

Sequence generation	16a	Method of generating the allocation sequence (eg, computer-generated random numbers), and list of any factors for stratification. To reduce predictability of a random sequence, details of any planned restriction (eg, blocking) should be provided in a separate document that is unavailable to those who enrol participants or assign interventions	Page 10 in Manuscript Protocol Version 5 Dated 20/07/2017

For peer review only - http://bmjopen.bmj.com/site/about/guidelines.xhtml

Allocation concealment mechanism	16b	Mechanism of implementing the allocation sequence (eg, central telephone; sequentially numbered, opaque, sealed envelopes), describing any steps to conceal the sequence until interventions are assigned	Page 10 in Manuscript Protocol Version 5 Dated 20/07/2017
Implementation	16c	Who will generate the allocation sequence, who will enrol participants, and who will assign participants to interventions	Page 10 in Manuscript Protocol Version 5 Dated 20/07/2017
Blinding (masking)	17a	Who will be blinded after assignment to interventions (eg, trial participants, care providers, outcome assessors, data analysts), and how	Page 12 in Manuscript Protocol Version 5 Dated 20/07/2017
	17b	If blinded, circumstances under which unblinding is permissible, and procedure for revealing a participant's allocated intervention during the trial	Page 12 in Manuscript Protocol Version 5 Dated 20/07/2017
**Methods: Data collection, management, and analysis**			
Data collection methods	18a	Plans for assessment and collection of outcome, baseline, and other trial data, including any related processes to promote data quality (eg, duplicate measurements, training of assessors) and a description of study instruments (eg, questionnaires, laboratory tests) along with their reliability and validity, if known. Reference to where data collection forms can be found, if not in the protocol	Page 10, 11 & 12 of manuscript & Protocol V5 dated 20/07/2017
	18b	Plans to promote participant retention and complete follow-up, including list of any outcome data to be collected for participants who discontinue or deviate from intervention protocols	Page 10 of manuscript Protocol V5 dated 20/07/2017
Data management	19	Plans for data entry, coding, security, and storage, including any related processes to promote data quality (eg, double data entry; range checks for data values). Reference to where details of data management procedures can be found, if not in the protocol	Protocol V5 dated 20/07/2017

For peer review only - http://bmjopen.bmj.com/site/about/guidelines.xhtml

Statistical methods	20a	Statistical methods for analysing primary and secondary outcomes. Reference to where other details of the statistical analysis plan can be found, if not in the protocol	Pages 13 & 14 of Manuscript and Protocol V5 dated 20/07/2017
	20b	Methods for any additional analyses (eg, subgroup and adjusted analyses)	Pages 13 & 14 of Manuscript and Protocol V5 dated 20/07/2017
	20c	Definition of analysis population relating to protocol non-adherence (eg, as randomised analysis), and any statistical methods to handle missing data (eg, multiple imputation)	Protocol V5 dated 20/07/2017
**Methods: Monitoring**			
Data monitoring	21a	Composition of data monitoring committee (DMC); summary of its role and reporting structure; statement of whether it is independent from the sponsor and competing interests; and reference to where further details about its charter can be found, if not in the protocol. Alternatively, an explanation of why a DMC is not needed	Pages 11 & 14 of Manuscript and Protocol V 5 dated 20/07/2017
	21b	Description of any interim analyses and stopping guidelines, including who will have access to these interim results and make the final decision to terminate the trial	Pages 11, 12 & 13 of Manuscript and Protocol V. 5 dated 20/07/2017
Harms	22	Plans for collecting, assessing, reporting, and managing solicited and spontaneously reported adverse events and other unintended effects of trial interventions or trial conduct	Pages 10 & 11. Protocol Version 5 Dated 20/07/2017
Auditing	23	Frequency and procedures for auditing trial conduct, if any, and whether the process will be independent from investigators and the sponsor	Pages 12 & 14 in Manuscript & Protocol V. 5 dated 20/07/2017

For peer review only - http://bmjopen.bmj.com/site/about/guidelines.xhtml

Ethics and dissemination

Research ethics approval	24	Plans for seeking research ethics committee/institutional review board (REC/IRB) approval	Page 3 in Manuscript Protocol Version 5 Dated 20/07/2017
Protocol amendments	25	Plans for communicating important protocol modifications (eg, changes to eligibility criteria, outcomes, analyses) to relevant parties (eg, investigators, REC/IRBs, trial participants, trial registries, journals, regulators)	Protocol V. 5 dated 20/07/2017
Consent or assent	26a	Who will obtain informed consent or assent from potential trial participants or authorised surrogates, and how (see Item 32)	Page 7 & 8 in Manuscript Protocol Version 5 Dated 20/07/2017
	26b	Additional consent provisions for collection and use of participant data and biological specimens in ancillary studies, if applicable	Page 7 & 8 of manuscript & Protocol V.5 dated 20/07/2017
Confidentiality	27	How personal information about potential and enrolled participants will be collected, shared, and maintained in order to protect confidentiality before, during, and after the trial	Protocol V.5 dated 20/07/2017
Declaration of interests	28	Financial and other competing interests for principal investigators for the overall trial and each study site	ICMJE Form for Disclosure of Potential Conflicts of Interest obtained from all involved and filed in main trial file. Non Disclosed to date.

For peer review only

For peer review only - http://bmjopen.bmj.com/site/about/guidelines.xhtml

Access to data	29	Statement of who will have access to the final trial dataset, and disclosure of contractual agreements that limit such access for investigators	MODEL AGREEMENT FOR NON-COMMERCIAL RESEARCH IN THE HEALTH SERVICE. Filed in Main trial file for all research sites involved
Ancillary and post-trial care	30	Provisions, if any, for ancillary and post-trial care, and for compensation to those who suffer harm from trial participation	Sheffield Teaching Hospital NHS Foundation Trust Indemnity applies
Dissemination policy	31a	Plans for investigators and sponsor to communicate trial results to participants, healthcare professionals, the public, and other relevant groups (eg, via publication, reporting in results databases, or other data sharing arrangements), including any publication restrictions	Page 14 in Manuscript Protocol Version 5 Dated 20/07/2017
	31b	Authorship eligibility guidelines and any intended use of professional writers	MODEL AGREEMENT FOR NON-COMMERCIAL RESEARCH IN THE HEALTH SERVICE. Filed in Main trial file for all research sites involved
	31c	Plans, if any, for granting public access to the full protocol, participant-level dataset, and statistical code	Protocol V.5 dated 20/07/2017

For peer review only - http://bmjopen.bmj.com/site/about/guidelines.xhtml

Appendices

Informed consent materials	32	Model consent form and other related documentation given to participants and authorised surrogates	Submitted with Protocol Version 5 Dated 20/07/2017 & manuscript
Biological specimens	33	Plans for collection, laboratory evaluation, and storage of biological specimens for genetic or molecular analysis in the current trial and for future use in ancillary studies, if applicable	Page 5 of manuscript,, Protocol V.5 dated 20/07/2017 & MODEL AGREEMENT FOR NON-COMMERCIAL RESEARCH IN THE HEALTH SERVICE. Filed in Main trial file for all research sites involved

*It is strongly recommended that this checklist be read in conjunction with the SPIRIT 2013 Explanation & Elaboration for important clarification on the items. Amendments to the protocol should be tracked and dated. The SPIRIT checklist is copyrighted by the SPIRIT Group under the Creative Commons "Attribution-NonCommercial-NoDerivs 3.0 Unported" license.

For peer review only - http://bmjopen.bmj.com/site/about/guidelines.xhtml
